# Indirect identification of genomic G-quadruplexes via a small protein probe that specifically recognizes C-rich single-stranded DNA

**DOI:** 10.1093/nar/gkag068

**Published:** 2026-01-30

**Authors:** Juan-nan Chen, Mei-lin Xie, Jiang-yu Yan, Ting-ting Cai, Yong-wen Ding, Tian-xiang He, Jiankang Wang, Jing Huang, Ke-wei Zheng

**Affiliations:** School of Biomedical Sciences, Hunan University, Changsha 410082, China; School of Biomedical Sciences, Hunan University, Changsha 410082, China; School of Biomedical Sciences, Hunan University, Changsha 410082, China; School of Biomedical Sciences, Hunan University, Changsha 410082, China; School of Biomedical Sciences, Hunan University, Changsha 410082, China; School of Biomedical Sciences, Hunan University, Changsha 410082, China; School of Biomedical Sciences, Hunan University, Changsha 410082, China; School of Biomedical Sciences, Hunan University, Changsha 410082, China; Hunan Provincial Key Laboratory of Animal Models and Molecular Medicine, Hunan University, Changsha 410082, China; School of Biomedical Sciences, Hunan University, Changsha 410082, China; Hunan Provincial Key Laboratory of Animal Models and Molecular Medicine, Hunan University, Changsha 410082, China

## Abstract

Detecting intracellular genomic G-quadruplexes (G4s) is crucial for understanding their biological functions. Although various G4 recognition probes have been developed, there remains a need for new G4 detection technologies to create detailed and reliable genomic G4 maps. In this study, we developed a small protein (CK13) that specifically recognizes the complementary C-rich single-stranded DNA (ssDNA) released during the formation of G4. Based on CK13 and CUT&Tag technology, we identified tens of thousands of C-rich ssDNA sites within human genomic DNA. These sites contain the vast majority of G4 sites detected by G4 probes, indicating that CK13 can well confirm the results of traditional G4 probes. Since CK13’s binding to C-rich ssDNA is minimally influenced by G4-binding proteins, it produces strong signals at the sites where intracellular G4-binding proteins are present. This indicates that, beyond free G4 structures, CK13 can also detect G4s occupied by G4-binding proteins within cells. Our findings demonstrate that C-rich ssDNA complementary to G4 can serve as an indirect marker for G4 formation, offering a promising approach to further explore the regulatory roles of G4s and their interacting proteins.

## Graphical Abstract

**Graphical Abstract:**
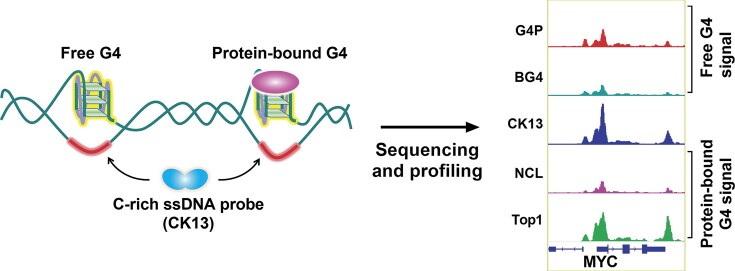

## Introduction

G-quadruplexes (G4s) are four-stranded secondary structures formed by guanine-rich nucleic acids. G4-forming sequences are widely distributed across the genomes of various organisms and are preferentially located in telomeres, gene promoters, replication origins, repeat regions, and satellite regions [1, 2], suggesting that they play a regulatory role in telomere maintenance [3, 4], gene transcription [5], DNA replication [6, 7], and recombination [8, 9].

G4s are key genomic structural features linked to cellular differentiation and cancers [10, 11]. However, the mechanisms by which G4s influence these processes remain to be fully understood. A major challenge in uncovering these mechanisms is monitoring the dynamic changes in G4 formation sites and frequencies within cells. Over the past 20 years, numerous fluorescent probes targeting G4s have been developed [12–18]. While some of these probes can quantitatively measure the overall G4 levels in living cells, they do not provide information about specific genomic G4 locations. To achieve genome-wide mapping, more sensitive detection methods integrating sequencing technologies have been developed, including high-throughput approaches based on G4 recognition proteins [19], G4 antibodies [20–22], and G4-specific chemical probes [23, 24]. These methods enable genome-wide mapping of G4s in genomic DNA and support the formation of G4 structures in living cells.

Most existing techniques for studying the genomic distribution of G4s rely on probes that bind specifically to G4 structures. The accuracy of these methods largely depends on the binding affinity, kinetics, and selectivity of the probes for G4s. Additionally, these probes must compete with intracellular G4-binding proteins for binding to G4 structures, which can lead to biased or incomplete detection of G4 sites [25]. G4access is a recently developed method that has the potential to overcome these limitations [26]. Its advantage is that it does not require G4-specific probes and can recognize G4 regardless of the presence of G4-binding proteins. However, this method may produce some false positive sites because it generates G-rich DNA fragments by enzymatic digestion in A/T-rich DNA regions, regardless of whether these sequences actually form G4 structures.

**Figure 1.**
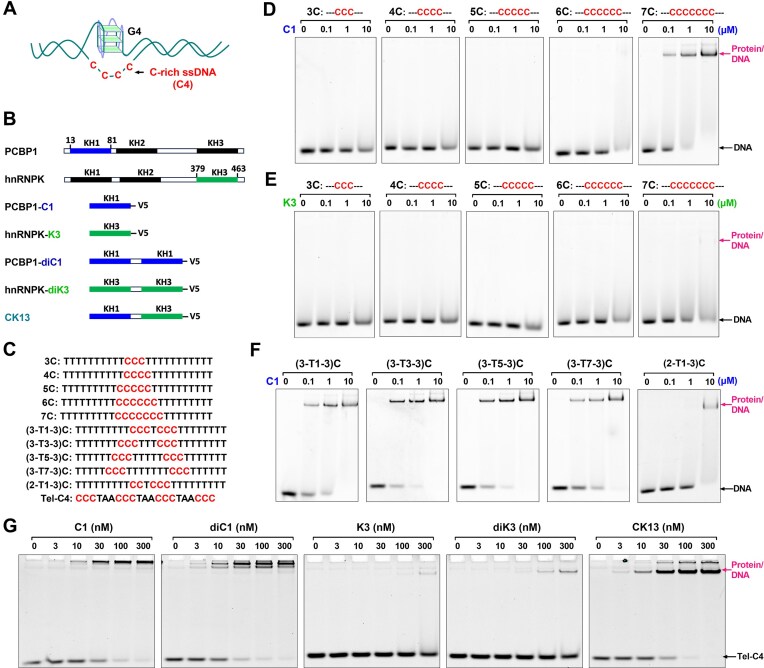
Exploration of protein probes that recognize C-rich ssDNA. (**A**) The formation of G-quadruplex (G4) in genomic DNA is accompanied by the release of its complementary C-rich ssDNA (C4). (**B**) Schematic diagram of the structural composition of poly(C) DNA binding protein. The three hnRNPK homology domains (KH1, KH2, and KH3) in PCBP1 and hnRNPK are all capable of binding poly(C) DNA. C1 and K3 are truncated constructs derived from the KH1 domain of PCBP1 and the KH3 domain of hnRNPK, respectively. diC1 and diK3 consist of two tandem repeats of KH1 or KH3 domains. CK13 is a chimeric protein combining the KH1 domain of PCBP1 and the KH3 domain of hnRNPK. All constructs are C-terminally fused with a V5 tag. (**C**) C-rich oligonucleotides used to assess protein–DNA binding affinity. (**D, E**) EMSA analysis of C1 and K3 binding to ssDNA containing tandem cytosine tracts of varying lengths. (**F**) EMSA analysis of C1 binding to discontinuous C-rich ssDNA. (**G**) EMSA comparison of the binding affinities of C1, K3, diC1, diK3, and CK13 to telomeric C-rich ssDNA (Tel-C4).

In genomic DNA, the formation of G4 is accompanied by the generation of C-rich single-stranded DNA (ssDNA) (Fig. 1A). Theoretically, the presence of these C-rich ssDNAs can serve as indirect evidence of G4 formation. However, there are currently far fewer methods available for detecting C-rich ssDNAs compared to those for detecting G4s. Previous studies have shown that C-rich ssDNAs can form i-motif structures, which typically require an acidic environment for stability [27–29]. An antibody called iMab was developed to detect intracellular i-motifs through methods such as iMab-IP-Seq [30, 31] and CUT&Tag [32], enabling the mapping of i-motif distribution across the genome. However, because i-motifs are more stable under acidic conditions, the binding affinity of iMab is strongly influenced by pH. In neutral or alkaline environments, this binding affinity is significantly reduced [29]. As a result, the use of iMab for intracellular i-motif detection may fail to identify many G4 formation sites that have not yet formed i-motif structures.

In this study, we developed a high-affinity probe, CK13, that selectively recognizes C-rich ssDNA complementary to G4s. CK13 is a 17 kDa protein composed of two DNA-binding domains derived from the human PCBP1 and hnRNPK proteins. *In vitro* experiments demonstrated that G4-binding proteins and CK13 can simultaneously bind to G4 structures and their complementary C-rich ssDNA, respectively, at the same G4-forming site within double-stranded DNA (dsDNA). Using CK13 in combination with CUT&Tag technology, we identified tens of thousands of C-rich ssDNA sites across the human genome. These sites largely overlapped with G4 sites detected by classical G4 probes and the binding sites of endogenous G4 recognition proteins, suggesting that CK13 is a powerful tool for mapping genomic G4s. Our study demonstrated that the presence of C-rich ssDNA complementary to G4 in genomic DNA can serve as indirect evidence for G4 formation.

## Materials and methods

### Materials

Oligonucleotides (Supplementary Tables S1–S3) were purchased from GENEWIZ (China) and Sangon Biotech (China). Anti-Flag M2 antibody (F1804) was purchased from Sigma–Aldrich. Anti-V5 tag antibody (ab15828), anti-Top1 antibody (ab109374), and anti-NCL antibody (ab70493) were purchased from Abcam. The coding sequences of C1, K3, di-C1, di-K3, and CK13 (Fig. 1B and Supplementary Table S4) were synthesized by Ruibio (Guangzhou, China) and inserted into pMAL-c5x vector (NEB) at the NdeI/BamHI restriction site. G4P and NCL were purified as previously described [19]. CUT&Tag kit for Illumina was obtained from Hunan Ruoyu Biotech (China).

### Protein expression and purification

The plasmids (pMAL-c5x-C1, pMAL-c5x-K3, pMAL-c5x-diC1, pMAL-c5x-diK3, pMAL-c5x-CK13) were transformed into *Escherichia coli* strain BL21. Cells were cultured in LB medium containing 50 µg/ml ampicillin at 37°C until OD_600_ was 1.0. Protein expression was induced with 0.8 mM isopropyl-β-d-1-thiogalactoside (IPTG) for 16 h at 16°C. Proteins were purified using Amylose Resin (NEB) according to the instructions in the product manual and digested with factor Xa protease (NEB) to remove MBP tag. The proteins were further purified using HiTrap Heparin HP (Cytiva) and stored in buffer containing 10 mM HEPES–KOH (pH 7.5), 150 mM NaCl, 50% glycerol, 1 mM ethylenediaminetetraacetic acid (EDTA), 1 mM DTT, 0.05% Triton X-100, and 1 mM AEBSF at −20°C.

### Preparation of fluorescently labeled proteins

The purified CK13, G4P, and NCL were diluted in PBS (pH 7.4) to a final concentration of 10 µM. Then, Cy3-NHS ester or Cy5-NHS ester (MCE) with a final concentration of 15 µM was mixed with the proteins and incubated at 4°C for 2 h. The labeled proteins were ultrafiltered using 10 kDa ultrafiltration tubes (Merck Millipore) to remove unreacted dyes and stored in buffer containing 10 mM HEPES–KOH pH 7.4, 300 mM NaCl, 50% glycerol (v/v), 1 mM EDTA, 1 mM DTT, and 0.05% Triton X-100 at −20°C.

### Electrophoretic mobility shift assay

5′-FAM labeled oligonucleotides (Supplementary Table S1) were dissolved at 20 nM in a buffer containing 20 mM Tris–HCl (pH 7.4), 75 mM KCl, 1 mM EDTA, 0.4 mg/ml BSA, denatured at 95°C for 5 min, and slowly cooled down to 25°C. DNA was then incubated with purified proteins at 4°C for 1 h. DNA samples were loaded on 10% non-denaturing polyacrylamide gel containing 75 mM KCl and electrophoresed at 4°C, 8 V/cm for 2 h in 1× TBE buffer containing 75 mM KCl. DNAs were visualized by the FAM dye covalently labeled at the 5′ end of the DNA on a Chemi-Doc MP (Bio-Rad). The dissociation constant (*K*d) was calculated using GraphPad Prism 6.0. The fraction of bound DNA (Y) was calculated by dividing the free DNA by the total DNA. Nonlinear regression analysis was performed using the equation: TXK = T0 + X + Kd, Y = [TXK - sqrt (TXK × TXK - 4 × T0 × X)]/(2 × T0), where T0 is the total DNA concentration and X is the total protein concentration.

For preparing dsDNA, oligonucleotides (Supplementary Table S3) were dissolved at 1 µM in buffer containing 10 mM Tris–HCl (pH 7.4), 75 mM LiCl, 1 mM EDTA, heated at 95°C for 5 min, and then cooled to 25°C at a rate of 0.1°C/s. dsDNAs were then diluted to 20 nM in buffer containing 10 mM Tris–HCl (pH 7.4), 75 mM KCl, 1 mM EDTA, 40% (v/v) PEG200, heated at 95°C for 5 min, and cooled down to 25°C at a rate of 0.1°C/s to allow G4 formation. The treated dsDNAs were incubated with G4P, NCL, or CK13 at 4°C for 1 h. DNA samples were loaded on 10% non-denaturing polyacrylamide gel containing 75 mM KCl, 40% (v/v) PEG 200 and electrophoresed at 4°C and 8 V/cm for 2 h in 1× TBE buffer containing 75 mM KCl. DNAs were visualized by the FAM dye covalently labeled at the 5′ end of the DNA on a Chemi-Doc MP (Bio-Rad).

### Circular dichroism spectroscopy

Oligonucleotides (Supplementary Table S1) were dissolved at 1.5 µM in buffers containing 20 mM Tris–acetate (pH 7.5, pH 6.5, or pH 5.5), 75 mM KCl, 1 mM EDTA, denatured at 95°C for 5 min, and cooled down to 25°C. In some samples, DNA was further incubated with 1.5 µM CK13 at 25°C for 1 h. Circular dichroism (CD) spectra were collected from 320 to 220 nm on a CD spectrophotometer (MOS-500, Bio-Logic, UK) at 25°C with 1-mm pathlength and 1-nm bandwidth. Buffer blank correction was made for all samples.

### Exonuclease digestion assay

The 5′-FAM labeled ssDNA (Ls-Tel-C4 and Ls-MYC-C4) (Supplementary Table S2) were dissolved at 0.05 µM in a buffer containing 40 mM Tris–HCl (pH 8.0), 50 mM KCl, 0.1 mM EDTA, heated at 95°C for 5 min, and cooled down to 25°C. Subsequently, DNAs were incubated on ice for 1 h in the presence of different concentrations of CK13. Prior to exonuclease digestion, MgCl_2_ and dithiothreitol (DTT) were added to the samples to reach final concentrations of 8 mM and 2 mM, respectively. The DNAs were then digested with 0.05 U/µl T4 DNA Polymerase (Thermo Scientific, USA) at 37°C for 5 min. Reactions were stopped by adding a final concentration of 50 mM EDTA and 0.1% SDS. Samples were heated at 95°C for 5 min in 80% formamide, separated on a 20% denaturing polyacrylamide gel, and visualized on a ChemiDoc MP (Bio-Rad).

### Cell lines and cell culture

HeLa and HCT116 cells were kindly provided by Stem Cell Bank, Chinese Academy of Sciences. Cells were cultured in DMEM supplemented with 10% FBS and 1% penicillin/streptomycin.

### Immunofluorescence staining

For detection of G4 and C-rich ssDNA, HeLa cells were seeded into 10 mm glass-bottom dishes and transiently transfected with plasmids encoding FLAG-tagged G4P and V5-tagged CK13 using Lipofectamine 3000 (Thermo Scientific, USA). After transfection, the cells were cultured for another 24 h, and then fixed with 4% paraformaldehyde and permeabilized with 0.5% Triton X-100 for 15 min at room temperature. The cells were blocked with 5% goat serum and 5% BSA in PBS overnight at 4°C before incubation with anti-V5 antibody (CST, D3H8Q) and anti-FLAG antibody (Sigma, F1804) for 1 h at 37°C. After three washes with PBST, secondary antibodies (Alexa Fluor 555 goat anti-rabbit IgG and Alexa Fluor 488 goat anti-mouse IgG, Invitrogen) were incubated with the cells at 37°C in the dark for another 1 h. Nuclei were stained with DAPI (5 μg/ml) and mounted with Fluoroshield mounting medium.

For detection of NCL colocalization with CK13, cells were transfected with plasmid encoding V5-tagged CK13 and subsequently incubated with anti-V5 antibody (CST, D3H8Q), anti-NCL antibody (CST, E5M7K), and secondary antibodies for 1 h at 37°C. Nuclei were stained with DAPI (5 μg/ml) and mounted with Fluoroshield mounting medium. Fluorescent imaging was performed on a Nikon CSU-W1 confocal laser scanning microscope.

### Cleavage under targets and tagmentation

The cleavage under targets and tagmentation (CUT&Tag) method was constructed following the described protocol [33] with some modifications. A total of 10^5^ cells were washed twice with 100 µl wash buffer (20 mM HEPES–KOH at pH 7.5, 150 mM KCl, 0.5 mM spermidine, 1× protease inhibitors). Next, 10 µl of pre-activated concanavalin A-coated magnetic beads were added to the cells in 100 µl wash buffer and incubated on a rotator for 10 min at room temperature. The supernatant was removed, and the bead-bound cells were resuspended in 100 µl of dig-wash buffer (0.05% digitonin, 0.1% BSA, 20 mM HEPES–KOH at pH 7.5, 150 mM KCl, 0.5 mM spermidine, 2 mM EDTA, 1× protease inhibitors) containing 250 nM of G4P, 200 nM of CK13, 2 µl of anti-NCL antibody (ab70493, Abcam), or 1 µl of anti-Top1 antibody (ab109374, Abcam), respectively. After 2 h of rotation at room temperature, the cells incubated with G4P or CK13 were washed twice with 100 µl of dig-wash buffer and resuspended with 100 µl of dig-wash buffer containing 2 µl of anti-FLAG M2 antibody (Sigma–Aldrich), or 2 µl of anti-V5 tag antibody (ab15828, Abcam) and incubated for another 1 h at room temperature. The buffer containing primary antibodies was then removed from the cells, and 100 µl of dig-wash buffer containing 1 µl of goat anti-mouse IgG (AP124, Sigma–Aldrich) or 1 µl of goat anti-rabbit IgG (AP132, Sigma–Aldrich) was mixed with the cells and incubated for 1 h at room temperature. After washing three times with dig-wash buffer, a 1:100 dilution of pAG-Tn5 adapter complex (∼0.2 µM) was prepared in 100 µl of dig-wash buffer and added to the cells, which were then rotated for 1 h at room temperature. After five washes with dig-wash buffer, the bead-bound cells were resuspended with 50 µl of tagmentation buffer [20 mM HEPES–KOH (pH 7.5), 150 mM KCl, 10 mM MgCl_2_, 0.5 mM spermidine, 1× protease inhibitors] and incubated at 37°C for 1 h. To terminate the tagmentation reaction, 2.5 µl of 0.5 M EDTA and 1 µl of 20 mg/ml proteinase K were added and incubated at 55°C for 30 min. The DNA was then extracted using 120 µl of Ampure XP beads (Beckman Coulter) for library preparation. DNA was eluted in 40 µl of 5 mM Tris–HCl (pH 8.0).

To quantify the library DNA, 2 µl of extracted DNA was mixed with an i5 and an i7 primer and amplified using Q5 High Fidelity 2× Master Mix (New England Biolabs) containing 1× SYBR green I. Real-time quantitative polymerase chain reaction (PCR) was performed on CFX Duet Real-Time PCR System (Bio-Rad). The cycling condition was 72°C for 5 min, 95°C for 3 min, and followed by 40 cycles of 30 s at 95°C and 30 s at 65°C. To amplify the libraries, 16 µl of DNA was mixed with a uniquely barcoded i5 and a uniquely barcoded i7 primer and amplified using Q5 high-fidelity 2× master mix (New England Biolabs). The PCR cycles for G4P and CK13 groups were 16 to 18, and those for Top1, NCL, and IgG groups were 15, 17, and 22, respectively. The libraries were purified with 1.3× volume of Ampure XP beads and eluted in 20 µl of 10 mM Tris−HCl (pH 8.0).

### CUT&Tag sequencing data analysis

The clean paired-end sequencing data were analyzed as described [12]. The human genome (version hg38) was used as a reference. Reads were aligned with bowtie2 [34] using --fast-local. Normalized (RPGC) coverage tracks were generated by deepTools [35] bamCoverage using parameters --binSize 50 --normalize --extendReads --ignoreDuplicates. Profiles and heatmaps of reads were generated from the bigwig files using the Deeptools computeMatrix followed by plotProfile and plotHeatmap, respectively, with region bed files derived from the NCBI RefSeq bed file downloaded from the UCSC website. Peaks of reads were identified with the MACS2 software [36] using --qvalue 0.001, --keep-dup 1, --nolambda. The putative G4-forming sequences (PQSs) of canonical G4 (4G), 4GL15 [37], GVBQ [38], and bulged G4 [39] were downloaded from GSE133379. The DeepTools multiBigwigSummary tool was used to calculate average scores across 300 bp genomic bins for each sample, excluding regions in the hg38 blacklist to avoid artifacts. Pairwise Pearson correlation coefficients were computed to assess sample similarity. The plotCorrelation tool was used to visualize the correlation matrix, with the --removeOutliers option enabled to exclude outliers. The overlaps between peaks from different samples were visualized using the Intervene upset (https://github.com/asntech/intervene).

## Results

### Construction of protein probes that recognize C-rich ssDNA

PCBP1 and hnRNPK are well-known C-rich ssDNA-binding proteins. They have three KH (K homology) domains (Fig. 1B), each comprising ~70 residues with a compact βααββα topology [40]. The three KH domains have different binding affinities for C-rich DNA. In PCBP1, the first KH domain (KH1) exhibits the highest affinity for C-rich DNA, followed by KH3 with moderate affinity, while KH2 has the weakest binding [41]. For hnRNPK, the KH3 domain shows the highest binding affinity for C-rich DNA [42]. Both truncated PCBP1-KH1 and hnRNPK-KH3 can bind C-rich DNA independently [41, 43]. Based on these natural C-rich DNA-binding proteins, it is promising to develop a mini-protein probe containing only the essential DNA-binding domains.

We first constructed and purified two protein probes: one containing the PCBP1-KH1 domain (designated C1) and the other containing the hnRNPK-KH3 domain (designated K3), each fused at the C-terminus with a V5 tag (Fig. 1B and Supplementary Fig. S1). We assessed their binding affinities to ssDNA sequences containing varying numbers of consecutive cytosine (C) bases using electrophoretic mobility shift assays (EMSA) (Fig. 1C). CD spectra showed that these DNAs were single-stranded in neutral solution (Supplementary Fig. S2). As shown in Fig. 1D, the C1 probe exhibited strong binding to ssDNA with seven consecutive cytosine bases. However, when the number of cytosines was reduced to six, binding affinity declined sharply, and significant binding was observed only at a high probe concentration (10 μM). In contrast, the K3 probe showed substantially weaker binding to the 7C sequence, and binding became nearly undetectable with fewer than seven cytosines (Fig. 1E). These results suggest that six or seven consecutive cytosines are the minimal binding units for effective interaction with C1 and K3 probes.

To determine whether every cytosine in the 7C stretch is necessary for binding, we introduced one or two base substitutions into the 7C DNA sequence. As shown in Fig. 1F, mutation of the central cytosine to generate the sequence CCCTCCC did not significantly affect C1 binding, suggesting the central base may act as a spacer and not contribute directly to binding. Since ssDNA can flexibly bend, an appropriate increase in the length of the spacer nucleotide in the middle of the C-rich sequence may have little effect on the binding of the DNA to the C1 probe. As expected, we found that the binding affinity of DNA to the C1 probe did not change greatly when the number of T bases in the middle of the CCCTCCC of the DNA strand was gradually increased from one to seven. However, when two cytosines in the C-rich region of 7C DNA were removed to yield a CCTCCC sequence, its interaction with the C1 probe was significantly weakened. These results indicate that strong interaction with the C1 probe requires at least six cytosines, allowing for a short, flexible spacer. The DNA recognition motif for the C1 probe can thus be summarized as CCCN_1_–_7_CCC.

The DNA sequence recognized by C1 probe closely matches the C-rich ssDNA complementary to G-quadruplex (G4) structures, typically consisting of four runs of G_3+_ tracts or three runs of G_3+_ tracts and one GG tract separated by loops of one to seven nucleotides [38, 39], [44, 45]. Therefore, these G4-associated C-rich ssDNAs (hereafter referred to as C4) in genomic DNA are ideal targets for C1 probe. Since C4 contains two copies of the sequences recognized by the C1 probe, to maximize the affinity of the probe for C4, we also prepared dimeric probes (diC1, diK3, and CK13) (Fig. 1B and Supplementary Fig. S1).

We evaluated the binding affinity of these probes to a C4 DNA derived from the human telomere (Tel-C4). As shown in Fig. 1G, the intensity of the band representing the original DNA (20 nM) decreased significantly, and a new band appeared in the gel well, suggesting that C1 effectively binds Tel-C4 but forms high-molecular-weight aggregates that hinder gel migration. The dimeric C1 probe (diC1) showed similar aggregation and no clear improvement in affinity. The dimeric K3 probe (diK3) showed slightly enhanced binding of Tel-C4 compared to the monomeric K3 probe and no significant aggregation of the DNA/protein complex, but its affinity for DNA was much lower than that of the C1 probe. The dimer probe (CK13) composed of C1 and K3 has a similar affinity for Tel-C4 as the monomeric C1 probe, and more importantly, a clear band of DNA/protein complexes was seen in the gel. This suggests that CK13 inherits the advantage of the high affinity of the C1 probe for C4 but is less prone to aggregation than C1.

### CK13 binds to C4 DNA with high affinity and selectivity

**Figure 2.**
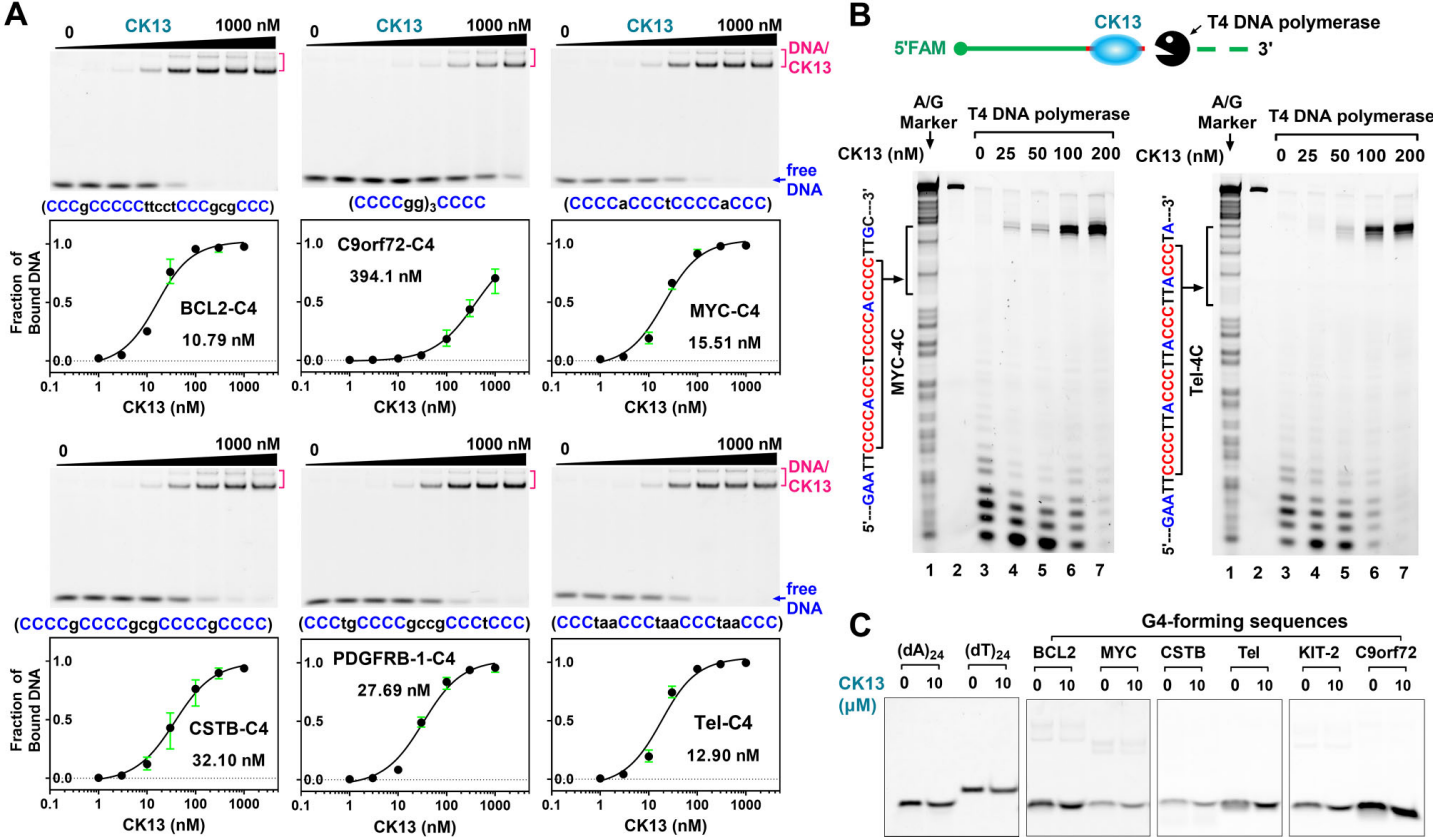
CK13 exhibits high affinity and specificity for C4 DNA complementary to G4 sequences in the human genome. (**A**) Binding affinity of CK13 to various C4 DNAs determined by EMSA. Each C4 contains four cytosine tracts and is derived from the complementary strand of a different G4-forming sequence. CK13 was tested at concentrations of 0, 1, 3, 10, 30, 100, 300, and 1000 nM. Dissociation constants (Kd, nM) were calculated accordingly. (**B**) CK13 protects the C4 region of ssDNA from 3′→5′ exonucleolytic degradation by T4 DNA polymerase. The marker in lane 1 indicates the positions of bases A and G. Sample 2 is the original DNA. Samples 3–7 were incubated with various concentrations of CK13 on ice for 1 h and then treated with 0.05 U/µl T4 DNA polymerase at 37°C for 5 min. (**C**) EMSA analysis showing that CK13 does not bind to (dA)_24_, (dT)_24_, and G-rich DNAs (G4-forming sequences).

To further validate CK13’s recognition of C4 DNA, we quantified its binding affinity for different types of C4s using EMSA. These C4s are complementary DNAs of six canonical G4s (BCL2 [46], C9orf72 [47], MYC [48], CSTB [19], PDGFRB-1 [49], and Tel [50]), two bulged G4s (EGFR [51] and KRAS [52]), two G-vacancy-bearing G4s (ABTB2 [38] and PDGFRB-2 [53]), and two 2-Quartet G4s (TBA [54] and KIT-1 [55]). CD spectra showed that these DNAs were mainly in ssDNA state under neutral conditions (Supplementary Fig. S3). As shown in Fig. 2A and Supplementary Fig. S4, CK13 binds strongly to most C4s containing two or more runs of C_3+_ tracts, with dissociation constants (Kd) ranging from 10.79 to 84.86 nM. An exception was C9orf72-C4, which exhibited a much weaker affinity (Kd = 394.1 nM). This is because C9orf72-C4 tends to form a hairpin structure [56], making it more difficult to bind to CK13. CK13 binds very weakly to TBA-C4 and KIT-1-C4 containing four CC tracts, which is consistent with the conclusion in Fig. 1 that the essential unit of the CK13 recognition sequence is CCCN_1_–_7_CCC.

We also tested the effect of base substitutions within the four cytosine tracts (C-tracts) of the C4 DNA (CD spectra was shown in Supplementary Fig. S5) on CK13 binding. As shown in Supplementary Fig. S6, mutating one C-tract significantly reduced the binding affinity of CK13 for MYC-C4 and Tel-C4. With two C-tract mutations, the binding affinity between them was further weakened, and detectable DNA–protein complexes were only observed at high CK13 concentrations (1 µM). When more than three C-tracts were mutated, CK13 was unable to bind the DNA. These findings indicate that while a minimum of two C-tracts is required for CK13 binding, the full complement of four C-tracts enables optimal interaction and maximizes binding affinity.

In addition to its strong affinity, CK13 also demonstrated high specificity for C4s. In exonuclease protection assays (Fig. 2B), CK13 selectively bound to the MYC-C4 and Tel-C4 regions within long ssDNA, protecting them from enzymatic degradation. EMSA results further confirmed this specificity: CK13 did not bind to poly(dA), poly(dT), or G-rich (G4-forming) sequences, even at concentrations up to 10 µM (Fig. 2C), underscoring its selective recognition of C-rich ssDNA.

**Figure 3.**
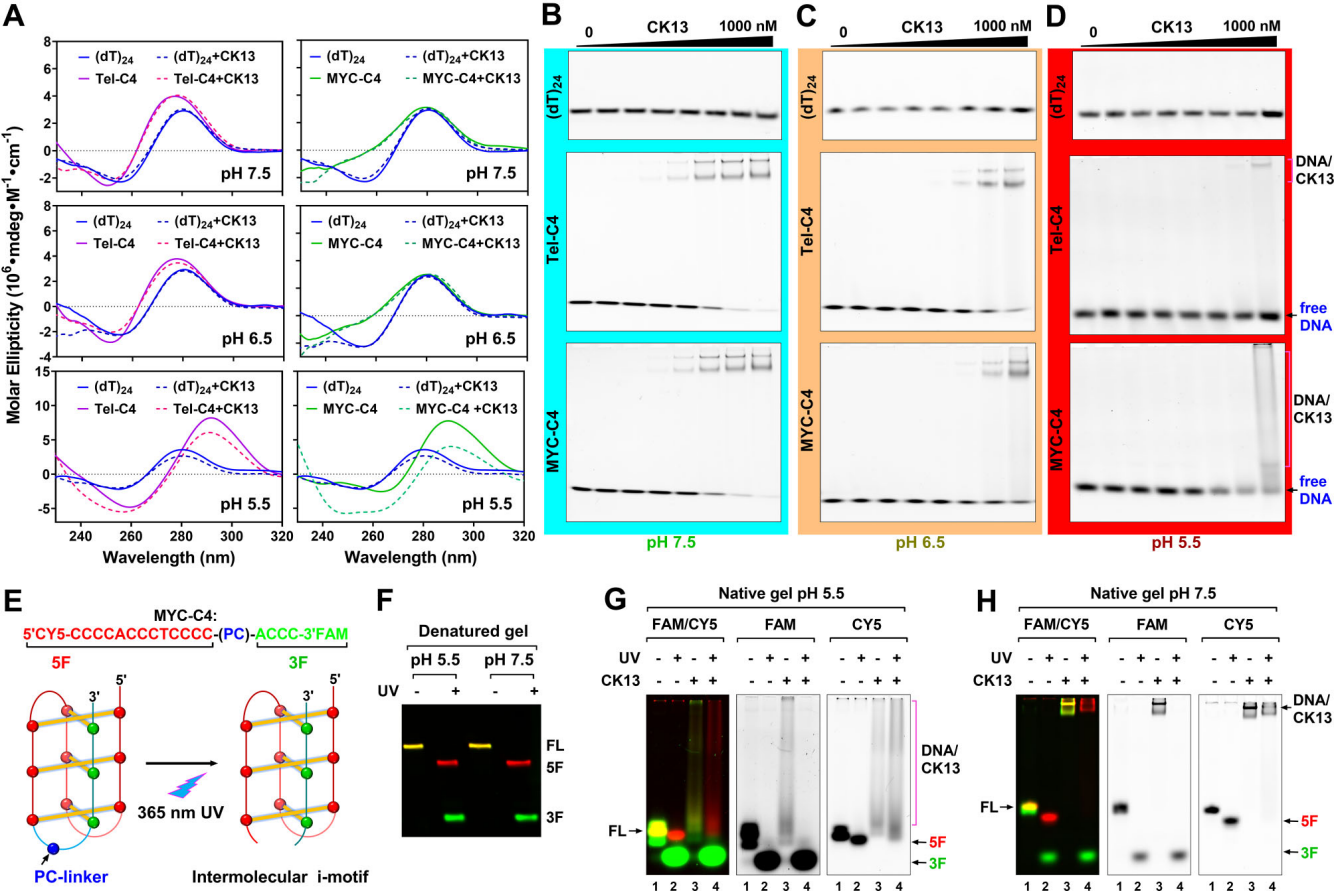
CK13 binds preferentially to unfolded C4 DNA rather than the i-motif structure. (**A**) CD spectra of (dT)_24_, Tel-C4, and MYC-C4 at pH 7.5, 6.5, and 5.5. DNA concentration was 1.5 µM. Dashed lines represent spectra of samples containing equimolar CK13 and DNA, after subtracting the signal of the buffer containing CK13 alone. (**B**–**D**) EMSA analysis of CK13 binding to (dT)_24_, Tel-C4, and MYC-C4 at pH 7.5, 6.5, and 5.5. CK13 was tested at gradient concentrations of 0, 1, 3, 10, 30, 100, 300, and 1000 nM. (**E**) Preparation of a uniform intermolecular i-motif. A modified MYC-C4 oligonucleotide was synthesized, containing a photocleavable (PC) linker between the third and fourth C-tracts, a CY5 label at the 5′ end, and a FAM label at the 3′ end. Upon 365 nm UV irradiation, the DNA cleaves at the PC linker, generating a Cy5-labeled fragment (5F) and a FAM-labeled fragment (3F). When the DNA adopts an i-motif structure, UV irradiation converts it into a uniform intermolecular i-motif. (**F**) Examination of the UV-cleavage efficiency of photocleavable MYC-C4 at pH 5.5 and pH 7.5 using denaturing gel electrophoresis. The full-length original DNA is marked as FL in the gel image. UV irradiation (365 nm) was performed for 1.5 min from the lid of the reaction tube at a distance of 0.5 cm. (**G, H**) EMSA analysis of CK13 binding to the intermolecular i-motif of MYC-C4 described in panel (E) at pH 5.5 and pH 7.5. The concentration of CK13 was 300 nM. The gel was scanned using the FAM and Cy5 channels, respectively.

Previous studies reported that C4s could form i-motif structures in cells, especially when the environmental pH was acidic [29, 31]. To assess whether CK13’s binding is dependent on i-motif formation, we first analyzed the CD spectra of MYC-C4 and Tel-C4 at different pH values, both in the presence and absence of CK13 (Supplementary Fig. S7). The results in Fig. 3A show that at pH 7.5 and 6.5, MYC-C4 and Tel-C4 exhibited absorption peaks similar to those of single-stranded (dT)_24_ DNA, and their CD spectra did not change significantly after the addition of CK13. When the pH value of the solution was adjusted to 5.5, the CD spectrum showed that MYC-C4 and Tel-C4 formed an i-motif structure, indicated by the positive peak at 288 nm and the negative peak at 260 nm. The positive peak signal at 288 nm of the C4 DNAs decreased slightly in the presence of CK13, which may be due to the unfolding of a small proportion of the i-motif structure by CK13. We further examined the binding affinity between CK13 and C4 at different pH using EMSA. The results in Fig. 3B show that CK13 exhibits strong binding to C4 at pH 7.5. Combined with the CD spectrum in Fig. 3A, we can infer that most of the DNA bound by CK13 at this pH is in a single-stranded state. When the pH value of the solution decreased from 7.5 to 6.5 and 5.5, the binding affinity of CK13 to DNA was significantly weakened (Fig. 3C and D). This may be due to the decrease in the binding ability of CK13 to DNA in an acidic environment, or the formation of i-motif hinders the binding of CK13 to DNA.

To further exclude the possibility that CK13 binds directly to the i-motif, we prepared an intermolecular i-motif structure and examined its interaction with CK13 (Fig. 3E). Theoretically, if CK13 recognizes and binds the i-motif, the folded structure should remain intact upon protein binding. In contrast, if CK13 binds single-stranded C-rich DNA, its binding would disrupt the i-motif, leading to unfolding and dissociation of the DNA strands forming the intermolecular structure.

The precursor sequence used to generate this intermolecular i-motif was MYC-C4, whose 5′ and 3′ ends were labeled with CY5 and FAM, respectively. A photocleavable (PC) linker was inserted between the third and fourth C-tracts (Fig. 3E). Upon irradiation with 365-nm UV light, the DNA cleaves at the PC linker, producing a Cy5-labeled large fragment containing three C-tracts and a FAM-labeled small fragment containing one C-tract (Fig. 3F). When MYC-C4 forms an i-motif structure, UV irradiation converts it into an intermolecular i-motif (Fig. 3E). The CD spectra in Supplementary Fig. S8A show that in a pH 5.5 solution, PC-linker–modified MYC-C4 exhibits a distinct positive peak near 288 nm, confirming the formation of an i-motif structure. After UV-induced cleavage, this positive signal is markedly reduced, indicating that the intermolecular i-motif cannot stably persist. This instability was further supported by non-denaturing gel electrophoresis, in which the CY5- and FAM-labeled fragments comprising the intermolecular i-motif became separated after UV irradiation (Fig. 3G). When incubated with CK13, only the Cy5-labeled large fragment displayed a mobility shift, indicating CK13 binding. The FAM-labeled small fragment, containing only one C-tract, did not bind CK13. The CD spectra of the intermolecular i-motif in Supplementary Fig. S8B further show that, in the presence of CK13, the positive peak near 288 nm is further reduced and shifts toward ~280 nm. These results demonstrate that CK13 does not bind or stabilize the intermolecular i-motif structure. At neutral pH (7.5), CK13 similarly showed no binding or stabilization of the intermolecular i-motif. Instead, it bound strongly to the Cy5-labeled single-stranded C-rich DNA component (Fig. 3H).

In summary, our data indicate that CK13 binds C4 DNA with high affinity and selectivity, and that its binding occurs when the DNA is in a single-stranded conformation rather than folded into an i-motif.

### CK13 and G4 recognition proteins can bind C4 and G4, respectively, at the same G4 formation site in dsDNA

In genomic dsDNA, the formation of G4 is accompanied by the release of its complementary C4 DNA. CK13 can indirectly demonstrate the formation of G4 by detecting C4. Furthermore, because CK13 and G4-binding proteins recognize different targets, CK13 may be less affected by the presence of intracellular G4-binding proteins. To investigate this possibility, we examined the binding of CK13 and G4-binding proteins to dsDNA that had undergone G4 formation.

**Figure 4.**
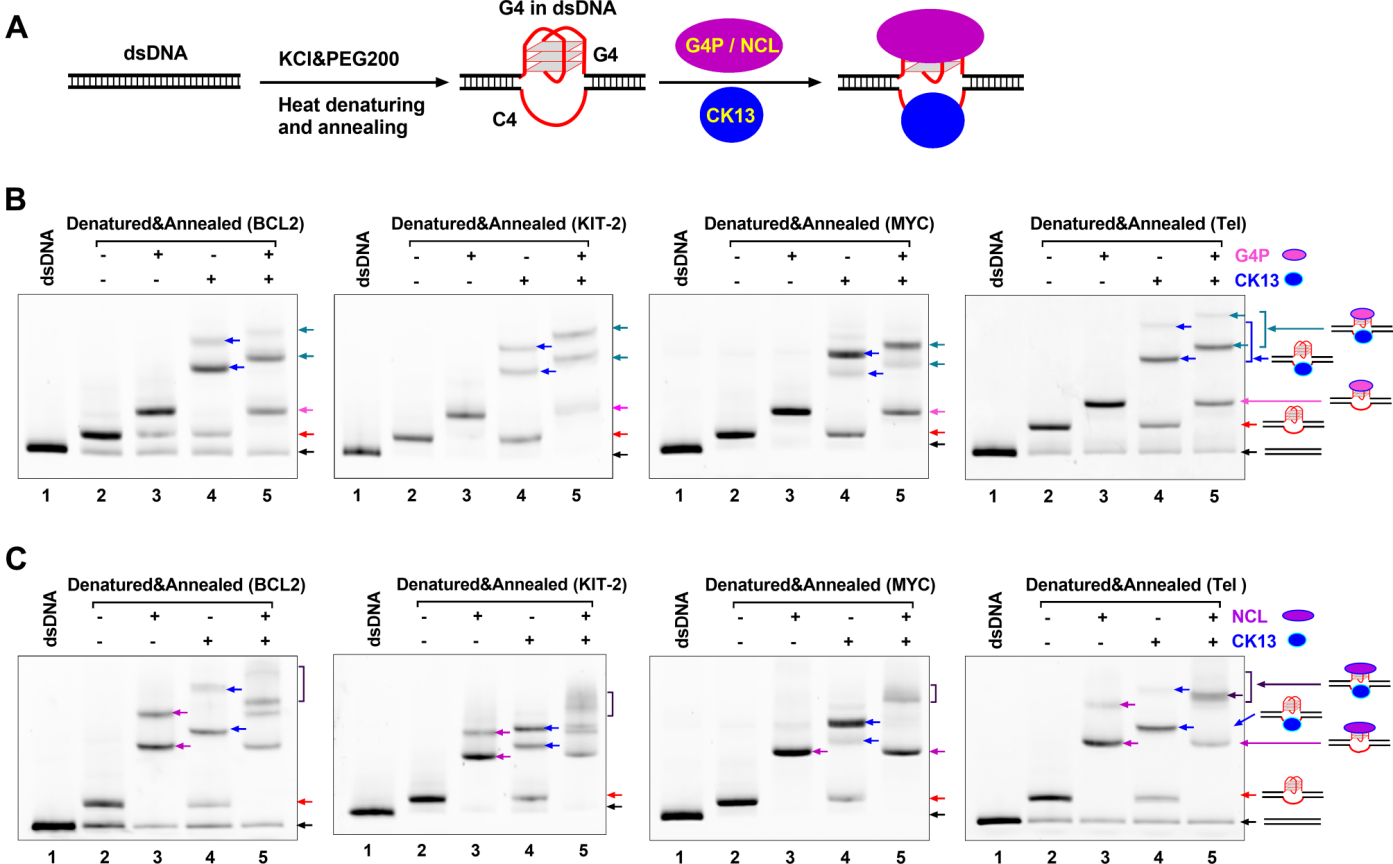
CK13 and G4-binding proteins simultaneously bind to C4 and G4 regions in dsDNA. (**A**) Schematic diagram of DNA–protein binding. The G-rich strand in dsDNA was 5′-end labeled with a FAM group. First, dsDNA was heat-denatured and annealed in 150 mM K⁺ with 40% (w/v) PEG 200 to promote G4 formation. Then, dsDNA that formed G4 (G4 in dsDNA) was incubated with 200 nM CK13, 200 nM G4P (or NCL), or 200 nM CK13 plus 200 nM G4P (or NCL) at 4°C for 1 h. Samples were resolved on 10% non-denaturing polyacrylamide gel containing 75 mM KCl and 40% (v/v) PEG200 at 4°C for 2 h in 1× TBE buffer containing 75 mM KCl. The gel was scanned on Chemi-Doc MP using the FAM channel. (**B**) EMSA analysis of CK13 and G4P binding to dsDNA that formed G4, prepared as in panel (A). Black arrow: unstructured dsDNA; red arrow: G4-structured dsDNA; pink arrow: G4P–DNA complex; blue arrow: CK13–DNA complex; cyan arrow: ternary CK13–G4P–DNA complex. (**C**) EMSA analysis of CK13 and NCL binding to dsDNA that formed G4, prepared as in panel (A). Purple arrow: NCL–DNA complex; blue arrow: CK13–DNA complex; brown arrow: ternary CK13–NCL–DNA complex.

We prepared four dsDNA substrates containing G4-forming sequences from human gene promoters (BCL2, KIT, and MYC) and telomeres (Tel), and subjected them to a heat denaturation and annealing protocol in the presence of 40% (v/v) PEG 200, following a previously reported method to promote G4 formation (Fig. 4A) [57]. In non-denaturing gel electrophoresis, the band corresponding to G4-containing DNA (Fig. 4B and C, red arrow) migrated more slowly than the original dsDNA band (black arrow), consistent with the structural change induced by G4 formation.

The G4-binding proteins used in this experiment include a G4 probe (G4P) and a truncated native G4-binding protein (NCL) [33, 58]. G4P is a small artificial G4-binding protein known for its specific recognition of G4s and its application in G4 detection using ChIP-seq and CUT&Tag [19, 33]. As shown in Fig. 4B, G4P binding to G4 in dsDNA produced a distinct shifted band corresponding to the G4P–G4 complex (pink arrow). CK13 binding to the complementary C4 strand resulted in two additional bands (blue arrows), which likely represent complexes with one or two CK13 molecules bound. When CK13 and G4P were added together, a slower-migrating band was observed (cyan arrow), suggesting the formation of a ternary complex in which both CK13 and G4P simultaneously bind to the same G4-forming region in the dsDNA. Similarly, binding of NCL to G4 generated two distinct shifted bands (Fig. 4C, purple arrows), likely reflecting one or two NCL molecules bound to G4. In the presence of both NCL and CK13, we observed additional slower-migrating bands (brown arrows), indicating the formation of ternary complexes containing NCL, CK13, and DNA. As a control group, dsDNA containing non-G4 sequences did not show any new electrophoretic bands after denaturation and renaturation, and was not bound by proteins (Supplementary Fig. S9).

To further verify that these slower migrating bands in Fig. 4B and C were complexes of DNA and proteins, we labeled CK13, G4P, and NCL proteins with CY3 or CY5 groups (Supplementary Fig. S10A), respectively, such that they could be visualized in the gel like DNA (labeled with FAM groups). The results in Supplementary Fig. S10B show that when CK13 or G4P binds to DNA, fluorescence signals of CY3 or CY5 can be detected in the shifted DNA bands, as expected. When CK13 and G4P bind to DNA simultaneously, fluorescence signals from the FAM, CY3, and CY5 groups appear in the slower migrating band, demonstrating that this band represents a ternary complex formed by DNA, CK13, and G4P. When Cy5-labeled NCL and Cy3-labeled CK13 bound to DNA simultaneously, a slower migrating band with three fluorescent signals was also detected, indicating that this band was a ternary complex formed by DNA, CK13, and NCL (Supplementary Fig. S10C).

In addition to the EMSA experiments described earlier, we also used immunofluorescence to examine whether CK13 can bind simultaneously with G4P or NCL to the G4 sites in human cells. We first transfected HeLa cells with plasmids encoding CK13 or G4P, each carrying a different tag for intracellular expression. The cellular localization of CK13 and G4P was then visualized using primary antibodies recognizing the tags and corresponding fluorescent secondary antibodies. NCL was identified using an antibody against the native NCL protein in cells. The results in Supplementary Fig. S11A show that in the nucleus, the fluorescence signal of G4P overlaps to a large extent with the fluorescence signal of CK13. A similar colocalization was also observed between NCL and CK13 (Supplementary Fig. S11B). These findings indicate that CK13 can bind concurrently with G4-recognition proteins at G4-forming sites in genomic DNA, further supporting its utility in detecting G4 structures independently and in combination with existing G4 probes.

### Detection of genomic G4 formation sites in human cells using CK13

Several chemical and protein-based probes that directly recognize G4s have been developed for detecting G4 structures in genomic DNA. However, alternative detection methods that do not rely on direct interaction with G4s remain limited. Targeting the C-rich ssDNA (C4), which is complementary to G4-forming sequences, offers a novel strategy for indirectly confirming G4 formation. This approach can complement and validate results obtained using conventional G4 detection techniques.

**Figure 5.**
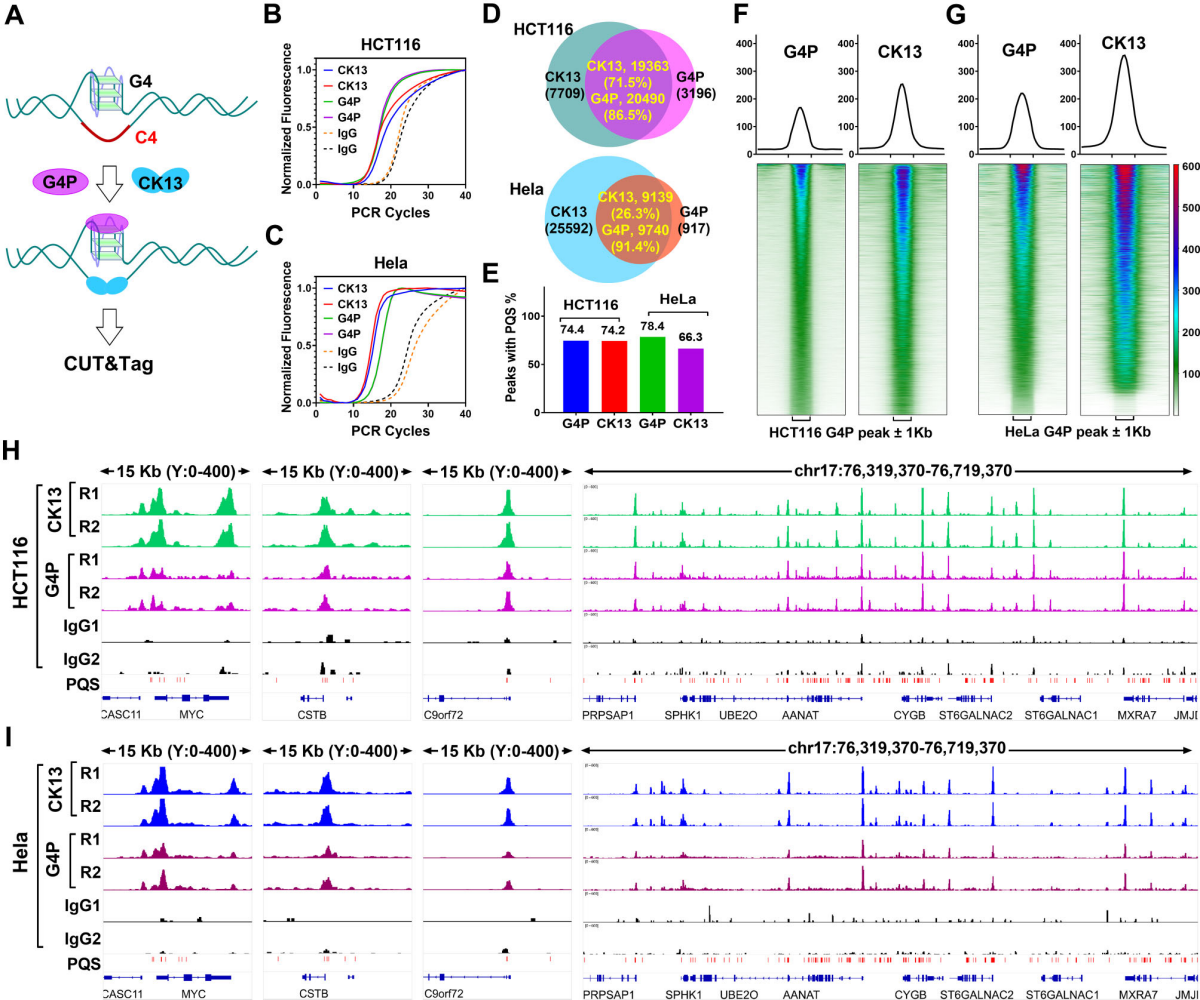
Colocalization of G4P and CK13 binding sites on genomic DNA. (**A**) Schematic illustration of CUT&Tag-based detection of G4P and CK13 binding sites in genomic DNA. (**B, C**) Real-time quantitative PCR analysis of DNA libraries prepared from HCT116 and HeLa cells via CUT&Tag. (**D**) Overlap of G4P and CK13 peak regions across the genome. (**E**) Proportion of G4P and CK13 peak regions overlapping with at least one putative G4-forming sequence (PQS) by one or more nucleotides. (**F, G**) Signal profiles (top) and heatmaps (bottom) of G4P and CK13 enrichment across ±1 kb regions centered on G4P peaks in HCT116 and HeLa cells. Heatmaps were sorted in descending order based on the mean G4P reads density. (**H, I**) Representative genomic loci showing colocalized binding of G4P and CK13 on chromatin DNA.

To evaluate this strategy, we employed the CUT&Tag method to detect genomic G4 and C4 regions in HCT116 and HeLa cells using G4P and CK13, respectively (Fig. 5A). G4P and CK13 were recognized by anti-Flag and anti-V5 antibodies, respectively, with normal mouse IgG serving as a negative control. Following labeling of genomic DNA with sequencing adapters via Tn5 transposase, the resulting libraries were amplified and quantified by qPCR using universal primers. As shown in Fig. 5B and C, the amplification curves for the G4P and CK13 groups appeared significantly earlier than those of the IgG control, indicating that a substantial number of DNA fragments were enriched through specific interactions with G4P or CK13.

We subsequently performed high-throughput sequencing, aligned the reads to the human reference genome, and conducted peak calling on the aligned data. As shown in Fig. 5D, 86.5% of G4P peaks overlapped with CK13 peaks in HCT116 cells, and this overlap ratio was 91.4% in HeLa cells. Furthermore, >66% of the peaks identified by both G4P and CK13 contained at least one putative G4-forming sequence (PQS) (Fig. 5E), consistent with prior observations using G4-specific probes [19].

To gain more insight into the colocalization of G4 and CK13, we sorted the G4P peak region file in descending order based on the mean G4P read counts and generated a heat map depicting the CK13 signal on the G4P peak region. The results in Fig. 5F and G show that the localization of CK13 and G4 is highly overlapped, and the signal intensity of CK13 is positively correlated with that of G4. Representative genome browser tracks in Fig. 5H and I further illustrate the striking similarity between G4P and CK13 binding profiles.

**Figure 6.**
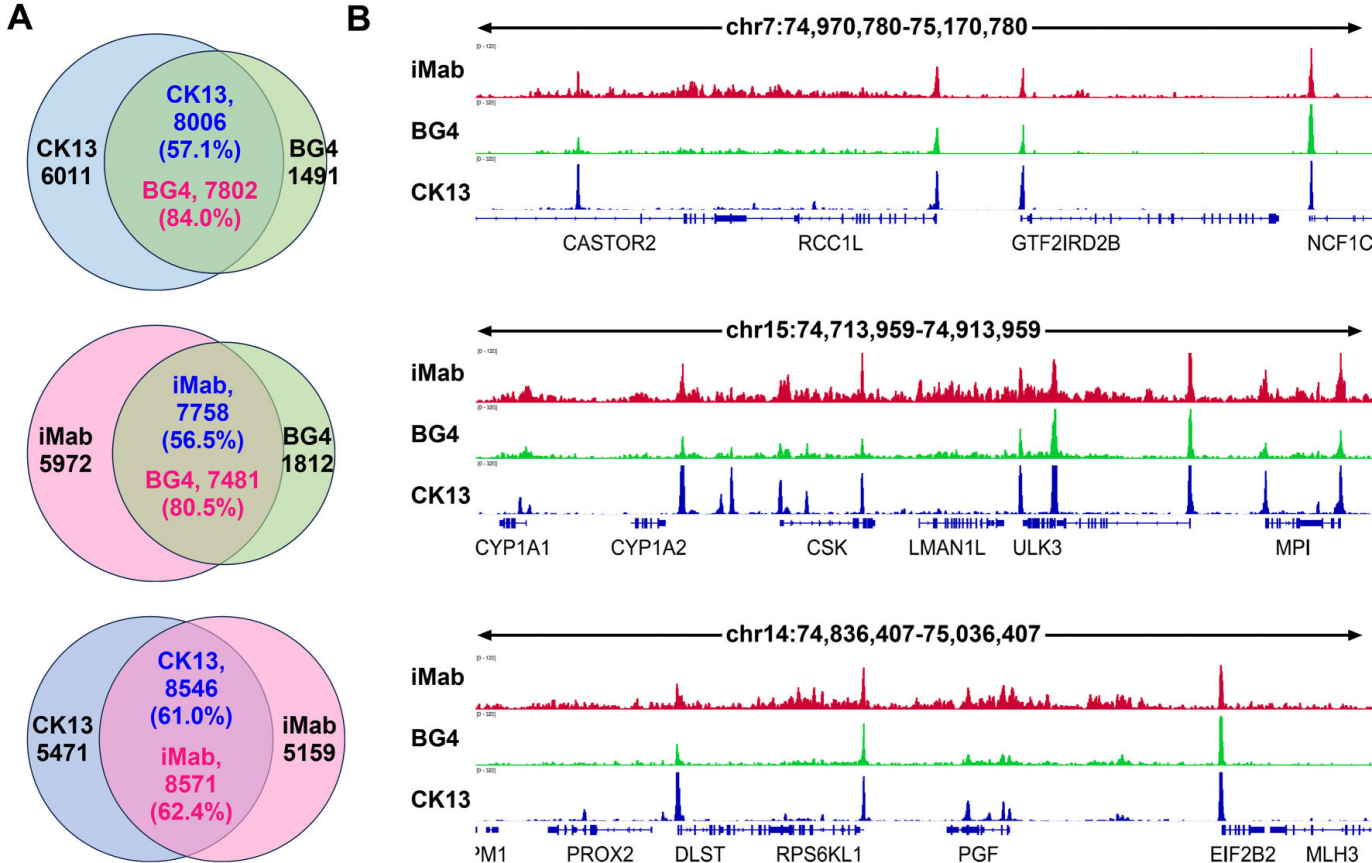
Comparison of CK13, BG4, and iMab binding sites on genomic DNA in HEK293T cells. CUT&Tag data for BG4 and iMab were downloaded from NCBI GEO under accession GSE220882. (**A**) Overlap of CK13, BG4, and iMab peak regions across the genome. Peak regions represent binding sites shared between replicate experimental groups. (**B**) Representative genomic loci showing CK13, BG4, and iMab binding signals in HEK293T cells.

We also compared the binding sites of CK13, BG4, and iMab on the genomic DNA of HEK293T cells. As shown in Fig. 6, CK13 and iMab share some similarities in their binding sites. For example, both probes cover >80% of BG4 binding sites, suggesting that they each recognize the majority of G4 formation sites. However, the direct overlap between CK13 and iMab binding sites is only ~60%. This discrepancy may arise from the fact that iMab recognizes i-motif structures, although this possibility cannot be conclusively evaluated at present due to the lack of independent tools for validating i-motif formation sites.

Beyond G4 formation, C-rich ssDNA may also arise during DNA replication or transcription. To evaluate whether non–G4-related processes contribute to CK13 binding signals, we classified PQS sequences in the human genome into two categories: those located within promoter regions [transcription start site (TSS) ± 3 kb] and those located outside promoters (Supplementary Fig. S12A). We then calculated the average CK13 signal distribution within 3 kb of the PQS centers. As shown in Supplementary Fig. S12B and C, similar to G4P, CK13 signals are highly enriched at PQS sites within gene promoters, while enrichment at PQS sites outside of gene promoter regions is minimal. These observations suggest that CK13 binding sites are predominantly located in gene promoter regions and are less associated with DNA replication–related events.

R-loop formation during transcription may contribute to the generation of C-rich ssDNA. To investigate the relationship between CK13 binding signals and R-loops, we treated cultured cells with ZnTTAPc, a compound known to modulate R-loop levels, and assessed CK13 binding sites and signal intensity using CUT&Tag. Previous studies have shown that ZnTTAPc can release Top1 proteins trapped on genomic G4s, enhance Top1 unwinding activity, and consequently reduce R-loop levels [59]. As shown in Supplementary Fig. S13A, compared with control cells, ZnTTAPc treatment reduced the mean R-loop signal at the TSS by ~40%. Conversely, the mean G4P and CK13 signals at the TSS were slightly increased after ZnTTAPc treatment, possibly due to ZnTTAPc’s ability to stabilize G4 [60, 61]. Representative genomic loci in Supplementary Fig. S13B further demonstrate that ZnTTAPc treatment broadly reduced R-loop signals without substantially altering G4P or CK13 signals. These results suggest that CK13 binding is not dependent on R-loop formation.

Together, these findings demonstrate that CK13 effectively identifies G4 formation sites across the genome and offers a reliable, indirect approach to G4 detection that is complementary to traditional G4-targeting probes.

### CK13 recognizes protein-bound G4

**Figure 7.**
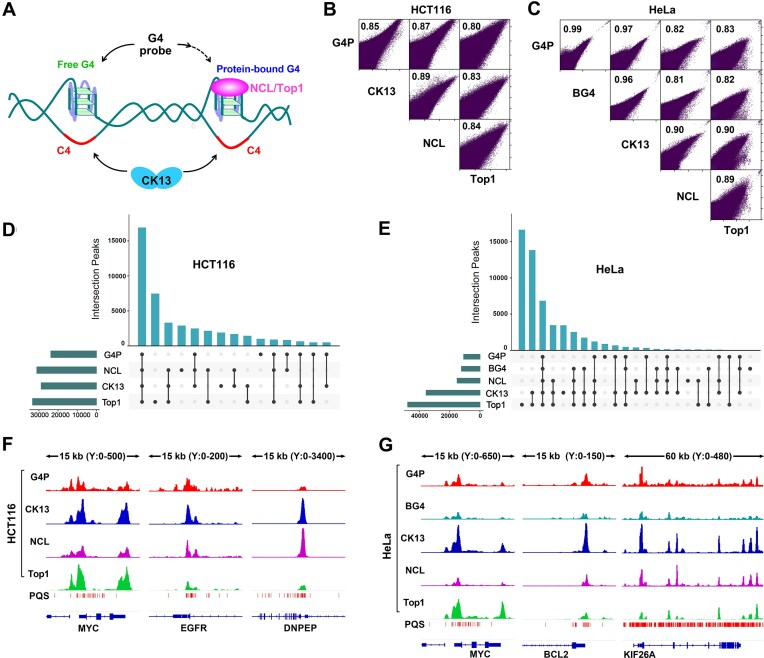
Colocalization of CK13 and endogenous G4-binding protein binding sites on genomic DNA. (**A**) The G4 probe preferentially binds to unoccupied G4s and has a more difficult time recognizing G4s that are already bound by endogenous G4-binding proteins. In contrast, CK13 is less affected by such protein occupancy. (**B, C**) Scatter plot matrices showing correlations between the signal intensities of G4 probes (G4P, BG4, CK13) and endogenous G4-binding proteins (NCL, Top1) in HCT116 and HeLa cells. Pearson correlation coefficients are indicated. (**D, E**) Overlap of peak regions between G4 probes and endogenous G4-binding proteins in HCT116 and HeLa cells. (**F, G**) Representative genomic regions showing G4-forming sites occupied by endogenous G4-binding proteins.

One challenge in detecting genomic G4s using G4-recognition probes is competition from endogenous G4-binding proteins, which can limit probe access to G4 sites and result in weak detection signals or failure to identify certain G4 loci (Fig. 7A). In contrast, CK13, which targets the G4-complementary C-rich ssDNA (C4), may be less affected by these endogenous proteins. This is supported by previous data showing that CK13 detects more binding sites than G4P (Fig. 5D) and exhibits stronger binding signals at G4-forming regions (Fig. 5F and G).

To further evaluate the potential advantages of CK13 in G4 detection, we applied CUT&Tag to map the binding profiles of two endogenous G4-binding proteins, nucleolin (NCL) and topoisomerase 1 (Top1), in HCT116 and HeLa cells. We then compared these profiles with those obtained using the G4 probes (G4P and BG4), as well as CK13. BG4 is a well-characterized single-chain variable fragment antibody specific to G4 structures and has been validated in ChIP-seq and CUT&Tag experiments [20, 62]. Pearson correlation analysis (Fig. 7B and C) revealed that the genomic binding profiles of NCL and Top1 were more strongly correlated with CK13 than with either G4P or BG4.

Further analysis of the peak regions showed that the number of CK13 binding sites closely matched those of NCL and Top1 and exceeded those detected by G4P and BG4. Among the three probes, CK13 also displayed the highest overlap in binding sites with both NCL and Top1 (Fig. 7D and E). These findings suggest that a portion of G4s in the genome may be preoccupied by native G4-binding proteins, rendering them less accessible to traditional G4-targeting probes such as G4P or BG4. In contrast, CK13 targets the G4-complementary C4 strand and, being unaffected by direct G4 occupancy, is capable of producing robust binding signals even at G4 sites bound by endogenous proteins (Fig. 7F and G).

## Discussion

Although several G4 recognition probes have been reported to detect G4 in genomic DNA ([13, 19, 20], [22–25]), their accuracy can be influenced by various factors, such as probe preferences for specific G4 conformations and competition with endogenous G4-binding proteins. To address the issues of probe’s recognition bias and nonspecific binding, different G4 detection technologies are often combined to cross-validate the results and improve reliability ([23], [25]).

In genomic dsDNA, G4 formation is accompanied by the release of complementary C-rich ssDNA, which serves as an indirect marker of G4 formation. There are two strategies for detecting the G4-complementary C-rich ssDNA in cells. The first is to detect i-motif structures formed by these sequences, for which the iMab antibody is a widely used tool [29]. Previous studies using iMab have demonstrated the presence of i-motifs in cells through immunostaining [29] and have identified genome-wide i-motif formation sites using CUT&Tag [30, 31, 32]. However, i-motif structures form most stably under acidic conditions [29], whereas the C-rich ssDNA released during G4 formation does not necessarily fold into an i-motif, especially in neutral physiological environments. As a result, probes targeting the i-motif may fail to recognize a subset of G4 formation sites.

The second strategy is to detect unfolded C-rich ssDNA, but this approach has not been reported previously. In this study, we developed a small protein probe, CK13, that selectively recognizes G4-associated C-rich ssDNAs. CK13 was engineered by combining the KH1 domain of PCBP1 with the KH3 domain of hnRNPK. Prior studies using systematic evolution of ligands by exponential enrichment (SELEX) [63], X-ray crystallography ([41], [64]), and nuclear magnetic resonance spectroscopy [65, 66, 67] have shown that PCBP1 KH1 and hnRNPK KH3 each specifically bind to CCC or CCCC sequences on ssDNA. Thus, CK13 should theoretically bind ssDNA containing two C_3+_ sequences. Our data confirmed this and further revealed that the linker between the two C_3+_ tracts can range from 1 to 7 nucleotides (Fig. 1F). However, longer linkers result in significantly reduced binding affinity between DNA and CK13 (Supplementary Fig. S6). This may be because the length of the peptide connecting the C1 domain and K3 domain in CK13 limits the interaction between the protein and DNA, which needs further investigation. CK13 exhibits high affinity and specificity for G4-complementary C-rich ssDNAs containing at least one C_3+_N_1_–_7_C_3+_ motif (Fig. 2 and Supplementary Fig. S4). CK13 binding sites and signal intensity on genomic DNA correlate strongly with those of G4P (Fig. 5 and Supplementary Fig. S12). These findings demonstrate that CK13 is a reliable tool for indirect identification of G4 formation.

Similar to G4P CUT&Tag [33] and BG4 CUT&Tag [62], CK13 CUT&Tag requires only ∼100 000 input cells and enables sequencing library preparation within ~12 h. Although CUT&Tag has inherent limitations (e.g. preference for open chromatin), it nonetheless allows CK13 to effectively detect tens of thousands of C-rich ssDNA formation sites across the genome and captures the majority of G4 formation sites identified by direct G4-binding probes (Figs 5D, 7D and E). Moreover, CK13 detects more genomic sites than direct G4-binding probes, and these sites exhibit higher overlap with binding sites of endogenous G4-binding proteins (Fig. 7D–G). This suggests that CK13 is capable of identifying more protein-bound G4 sites than conventional G4 probes. By integrating data from both G4 probes and CK13, it is promising to distinguish between free and protein-bound G4s in different cell types or developmental stages. In addition, comparison of CK13 and iMab sequencing data enables more accurate determination of whether C-rich DNA at G4 sites folds into an i-motif or remains single-stranded, and allows quantification of the proportion of DNA forming i-motifs. Such information is crucial for understanding the functional roles and mechanisms of these secondary DNA structures in transcriptional regulation.

Despite its advantages, several potential limitations should be considered when using CK13 to detect G4-forming sites. First, partially unfolded DNA generated during replication or transcription could lead to CK13 binding at G4-independent regions. However, replication and transcription dynamics vary among individual cells, resulting in heterogeneous locations of replication or transcription bubbles. Even if CK13 binds to such bubbles in a fraction of cells, the averaged signal across the population would be diluted. Consistent with this, our data show that CK13 signals are highly enriched at PQS sites within gene promoters, while enrichment at PQS sites outside of gene promoter regions is minimal (Supplementary Fig. S12). This suggests that partially unfolded DNA arising from replication or transcription has limited impact on CK13-based detection of G4 formation sites. Second, R-loops may influence CK13 recognition. During transcription, R-loops preferentially form when the non-template strand is G-rich [68, 69]. If the G4-complementary C-rich ssDNA engages in an RNA:DNA hybrid, CK13 would be unable to access or detect that site. However, treatment of cells with compounds that reduce R-loop levels did not decrease the CK13 signal intensity (Supplementary Fig. S13), indicating that the effect of the R-loop on CK13 binding is not significant. Third, endogenous ssDNA-binding proteins or C-rich DNA-binding proteins may compete with CK13 for target sites. When these proteins are present at high abundance, they can occupy C-rich ssDNA, potentially reducing the number of sites detected by CK13. In summary, while CK13 is a powerful tool for indirect identification of G4 formation sites, there remains the possibility of false positives or false negatives. Validation using complementary methods is therefore recommended.

Beyond G4 detection, CK13 can also be used to label native proteins that interact with G4s *in situ*. Similar strategies have been reported using chemical [70, 71] or protein-based probes [72] targeting G4. Compared to G4 probes, CK13 has a higher probability of binding to the same G4 site with native G4-binding protein simultaneously. Therefore, CK13 is expected to identify more unknown G4-interacting proteins in the future.

## Supplementary Material

gkag068_Supplemental_File

## Data Availability

Top1 CUT&Tag data of HCT116 cells were downloaded from the NCBI Gene Expression Omnibus (GEO) under accession code GSE239694. G4P and BG4 CUT&Tag data of HeLa cells were downloaded from the NCBI GEO under accession code GSE270287. The file containing PQS information was downloaded from the NCBI GEO under accession code GSE133379. The CUT&Tag data of HCT116 cells (G4P, CK13, and NCL), HEK293T cells (CK13), and HeLa cells (CK13, Top1, and NCL) have been deposited to the NCBI GEO under accession code GSE296647.
