# Supplementary material for: Indirect identification of genomic G-quadruplexes via a small protein probe that specifically recognizes C-rich single-stranded DNA
Source: Nucleic Acids Res. 2026 Jan 30;54(3):gkag068. doi: 10.1093/nar/gkag068 (PMC12856213; doi:10.1093/nar/gkag068)
Supplement: gkag068_Supplemental_File [file gkag068_supplemental_file.pdf]

Supporting Information for

Indirect identification of genomic G-quadruplexes via a small protein probe that specifically recognizes C-rich single-stranded DNA

Juan-nan Chen[1], Mei-lin Xie[1], Jiang-yu Yan[1], Ting-ting Cai[1], Yong-wen Ding[1], Tian-xiang He[1], Jiankang Wang[1], Jing Huang[1, 2, *], Ke-wei Zheng[1, 2, *]

[1]School of Biomedical Sciences, Hunan University, Changsha 410082, China

[2]Hunan Provincial Key Laboratory of Animal Models and Molecular Medicine, Hunan University, Changsha, China

*Correspondence should be addressed to Jing Huang (huangjing16@hnu.edu.cn) or Ke-wei Zheng (zhengkewei@hnu.edu.cn)

Supplementary Figures S1-S13, Tables S1-S4.

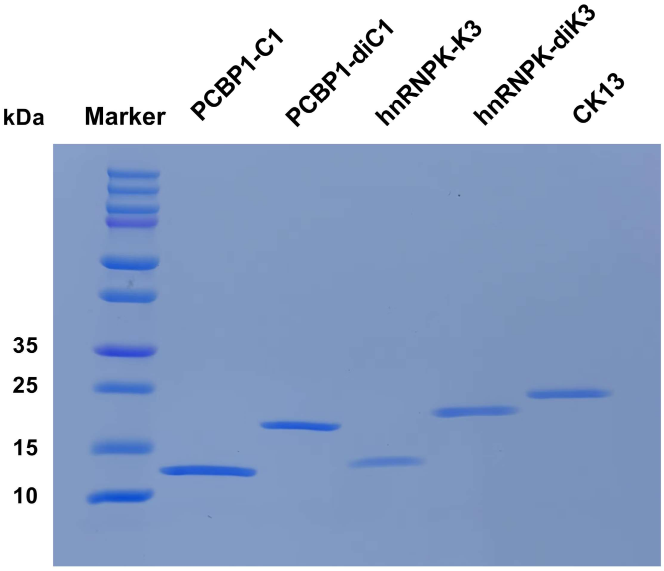

Figure S1 Purified recombinant proteins. Gel was stained by Coomassie brilliant blue G250.

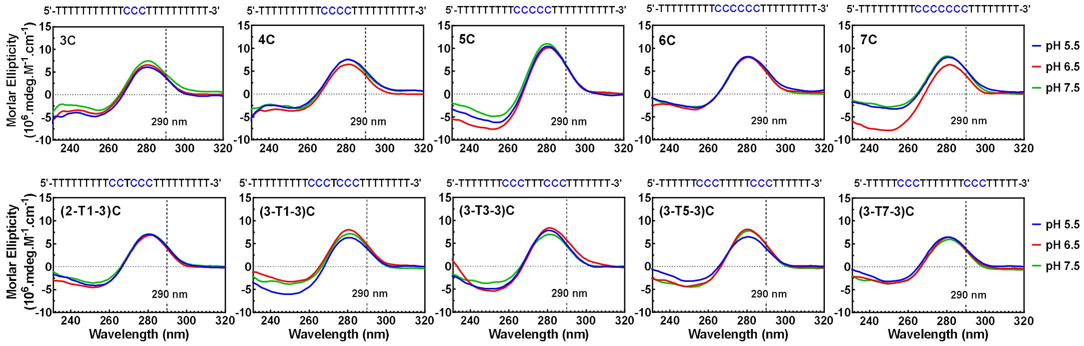

Figure S2. Circular dichroism (CD) spectra of C-rich single-stranded DNAs at pH 5.5, 6.5, and 7.5.

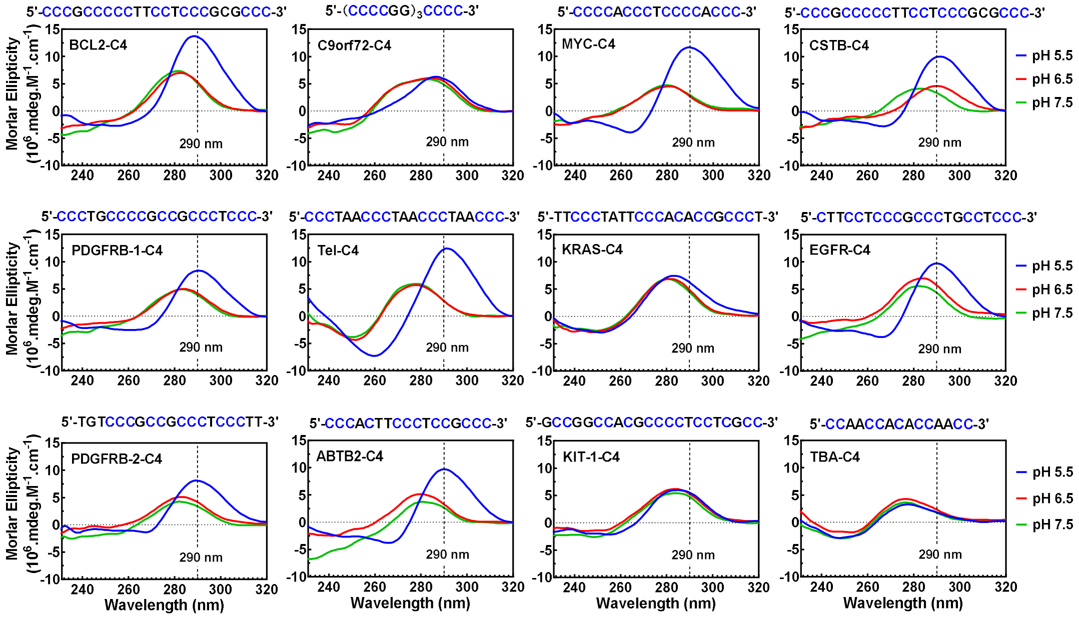

Figure S3. Circular dichroism (CD) spectra of C4 DNAs at pH 5.5, 6.5, and 7.5.

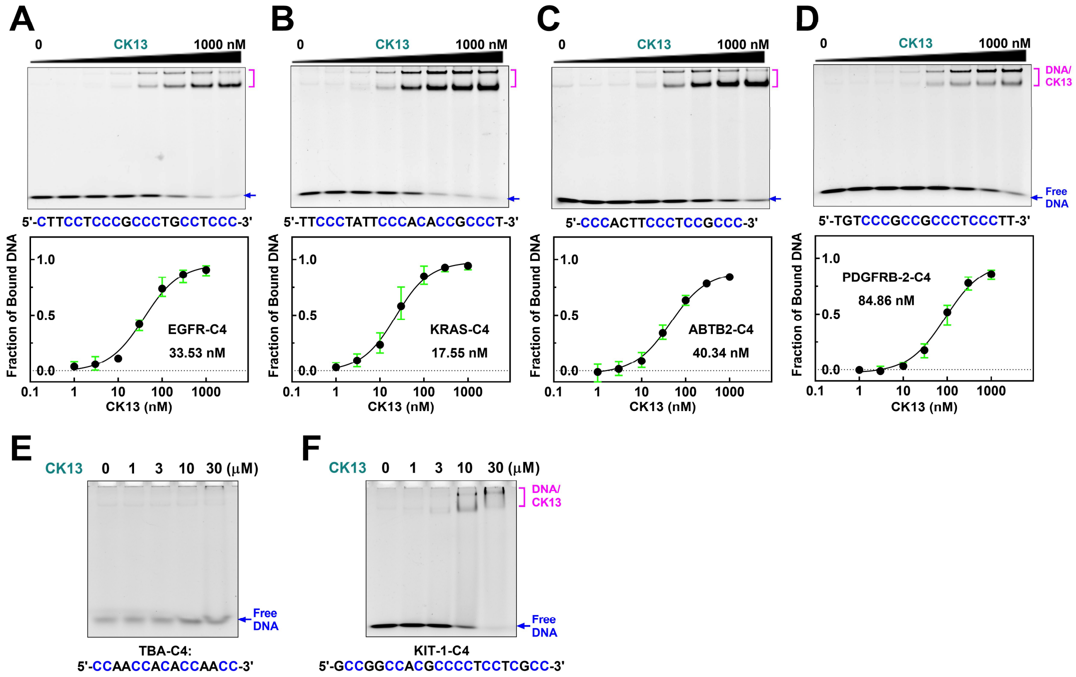

Figure S4. (A-D) Binding affinity of CK13 to various C4 DNAs determined by EMSA. Each C4 contains four cytosine tracts and is derived from the complementary strand of a different G4-forming sequence. CK13 was tested at concentrations of 0, 1, 3, 10, 30, 100, 300, and 1000 nM. Dissociation constants (Kd, nM) were calculated accordingly. (E-F) EMSA analysis of CK13 binding to TBA-C4 and KIT-1-C4, which are complementary sequences of 2-Quartet G4s. CK13 was tested at concentrations of 0, 1, 3, 10, 30 μM.

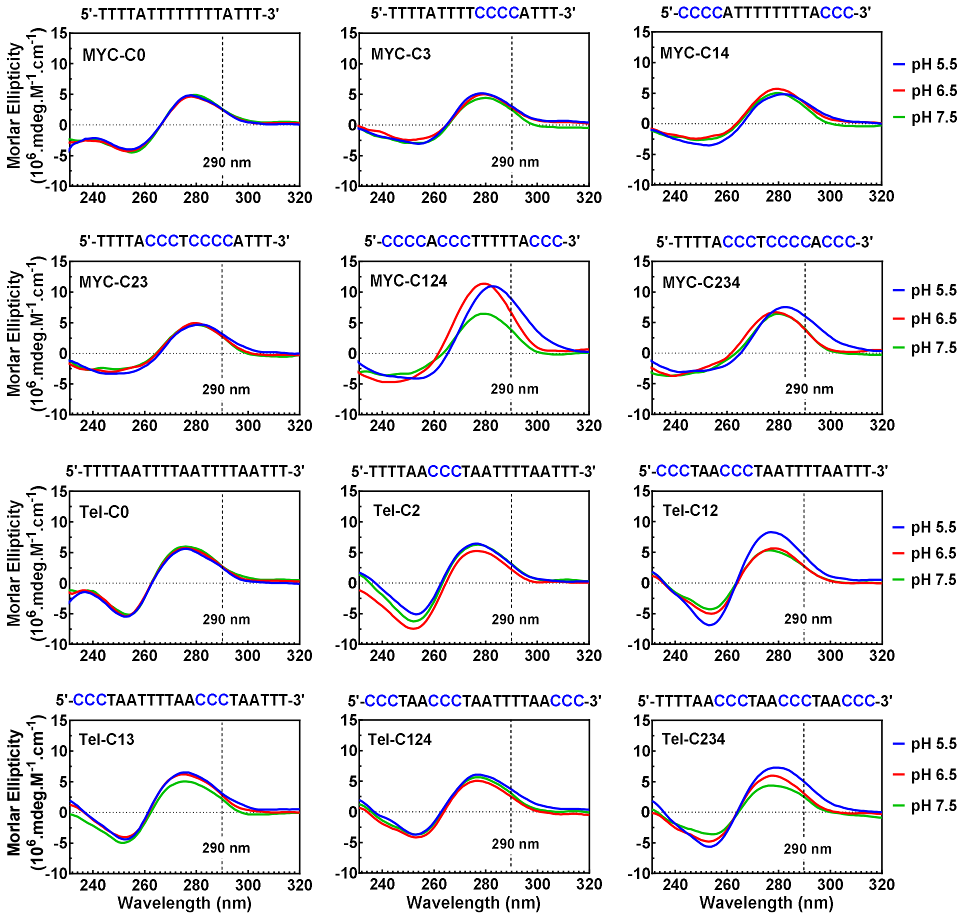

Figure S5. Circular dichroism (CD) spectra of mutant MYC and telomere (Tel) C-rich ssDNA at pH 5.5, 6.5, and 7.5.

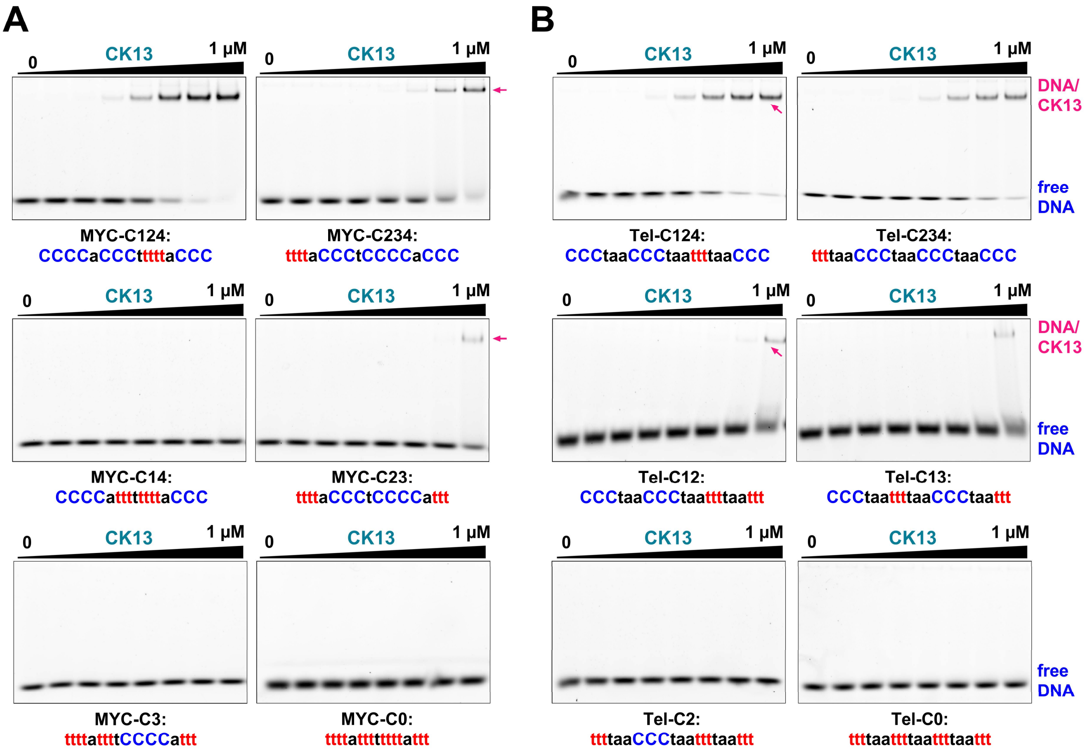

Figure S6. (A-B) Detection of the binding ability of mutant MYC and telomere (Tel) C-rich ssDNA to CK13 using EMSA. The C-tract (blue font) in the mutated DNA is replaced by poly(dT) (red font). CK13 was tested at concentrations of 0, 1, 3, 10, 30, 100, 300, and 1000 nM.

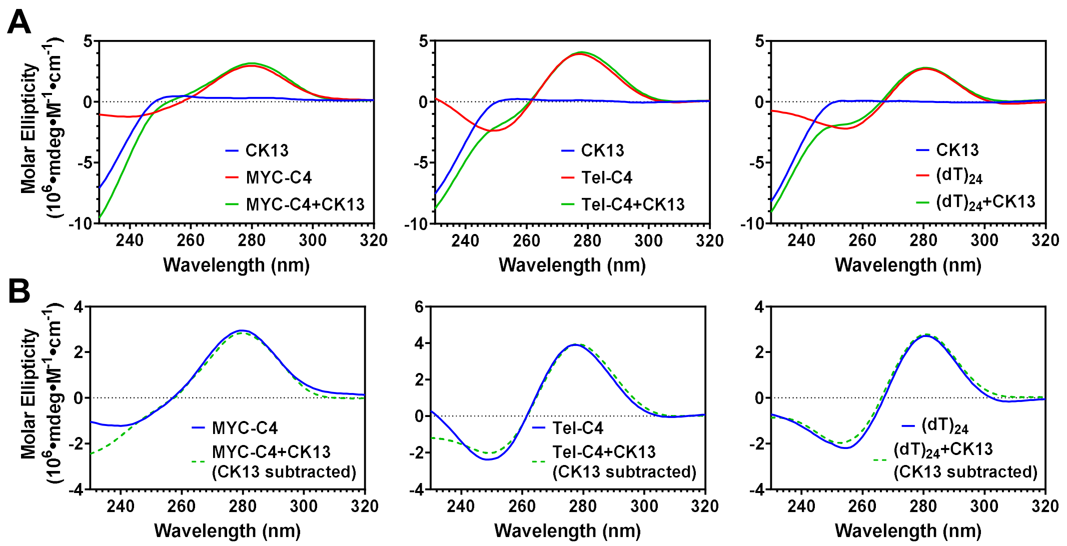

Figure S7. (A) Circular dichroism (CD) spectra of CK13 and DNAs ($(dT)_{24}$, Tel-C4, and MYC-C4) in the presence or absence of CK13 at pH 7.5. CK13 and DNA concentration was 1.5 μM. (B) Comparison of CD spectra of DNAs in the presence or absence of CK13. The dashed green line shows the spectrum of the sample containing CK13 and DNA after subtraction of the signal of the sample containing CK13 alone.

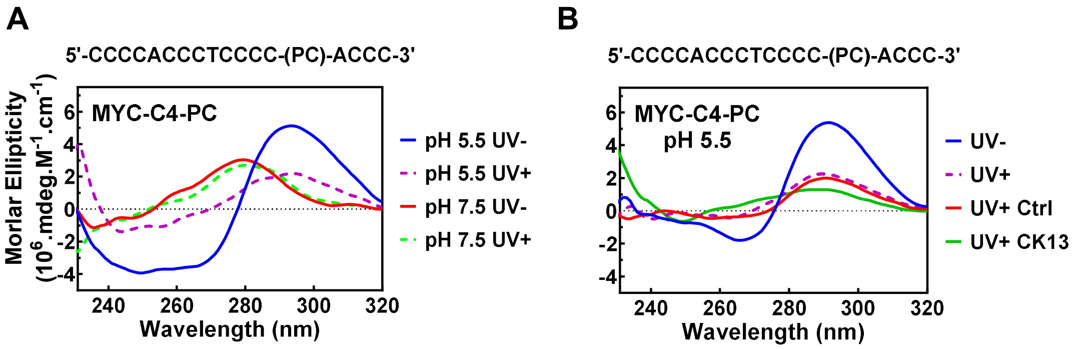

Figure S8. (A) Circular dichroism (CD) spectra of photocleavable (PC) linker-modified MYC-C4 before (UV-) and after (UV+) UV irradiation at pH 5.5 and 7.5. (B) The unfolding effect of CK13 on the intermolecular i-motif structure of MYC-C4. After UV irradiation at 365 nm for 1.5 min at pH 5.5, the PC linker–modified MYC-C4 was incubated at room temperature for 3 hours in the absence (Ctrl) or presence of CK13 (2.0 molar equivalents relative to MYC-C4). CD spectra of MYC-C4 were then acquired. For samples containing both CK13 and DNA, the CD signal of CK13 alone was subtracted from the measured spectrum.

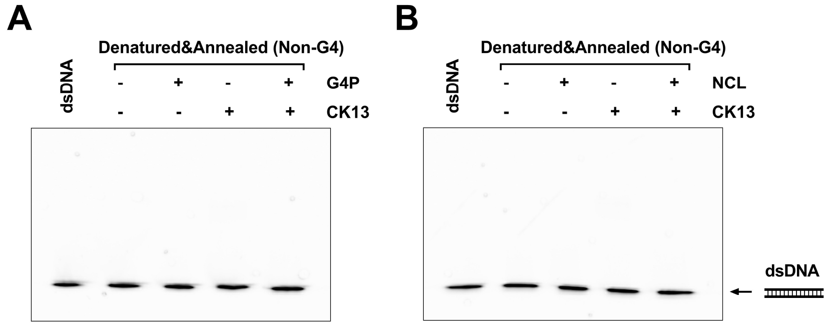

Figure S9. (A-B) EMSA analysis of CK13, G4P, and NCL binding to dsDNA with sequences that do not form G4 (non-G4). As described in Figure 4, dsDNA was heat-denatured and annealed in 150 mM K^+ with 40% (w/v) PEG 200. Then, DNAs were incubated with 200 nM CK13, 200 nM G4P (or NCL), or 200 nM CK13 plus 200 nM G4P (or NCL) at 4℃ for 1 hour. Samples were resolved on 10% non-denaturing polyacrylamide gel containing 75 mM KCl and 40% (v/v) PEG200 at 4 ℃ for 2 hours in 1× TBE buffer containing 75 mM KCl. The gel was scanned on Chemi-Doc MP using the FAM channel.

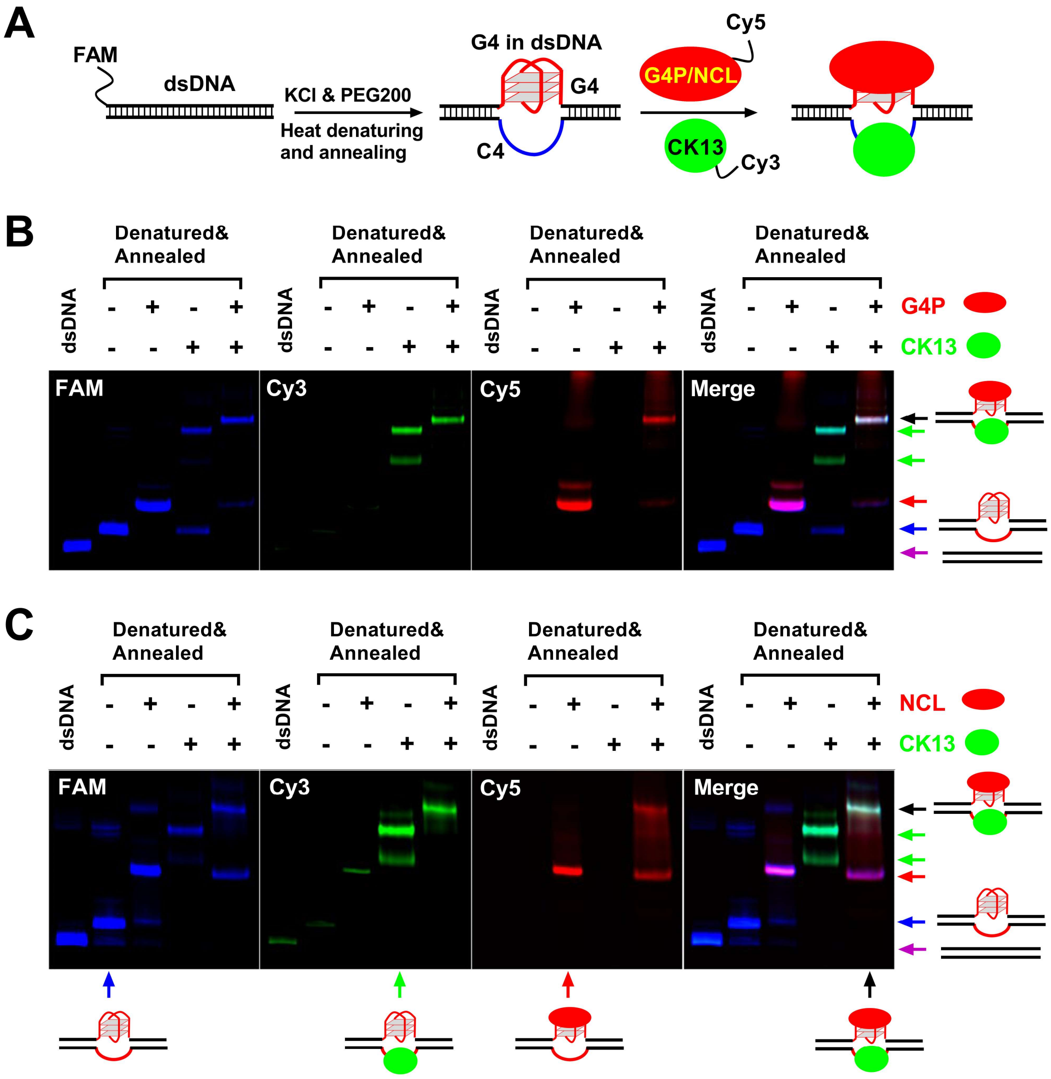

Figure S10. Verification of the recognition of C4 and G4 in dsDNA by CK13 and G4-binding protein using fluorescently labeled DNA and proteins. (A) Schematic diagram of DNA-protein binding. The G-rich strand in dsDNA was 5′-end labeled with a FAM group. CK13 was labeled with Cy3 groups. G4P and NCL were labeled with Cy5 groups. Firstly, dsDNA was heat-denatured and renatured in 150 mM K^+ with 40% (w/v) PEG 200 to promote G4 formation (1). Then, dsDNA that formed G4 (G4 in dsDNA) was incubated with 200 nM CK13, 200 nM G4P, or 200 nM CK13 plus 200 nM G4P at 4℃ for 1 hour. Samples were resolved on 10% non-denaturing polyacrylamide gel containing 75 mM KCl and 40% (v/v) PEG 200 at 4 ℃ for 2 hours in 1× TBE buffer containing 75 mM KCl. The gel was scanned on Chemi-Doc MP using the FAM, Cy3 and Cy5 channels respectively. (B) EMSA analysis of CK13 and G4P binding to dsDNA containing MYC G4. FAM, Cy3, and Cy5 represent images displayed by three fluorescence channels. The merged image is an overlay of images from the FAM, Cy3, and Cy5 channels. Purple arrow: linear dsDNA; blue arrow: dsDNA that formed G4 (G4 in dsDNA); red arrow: G4P—DNA complex; green arrow: CK13—DNA complex; Black arrow: ternary CK13—G4P—DNA complex. (C) EMSA analysis of CK13 and NCL binding to dsDNA containing MYC G4. Purple arrow: linear dsDNA; blue arrow: dsDNA that formed G4 (G4 in dsDNA); red arrow: NCL—DNA complex; green arrow: CK13—DNA complex; Black arrow: ternary CK13—NCL—DNA complex.

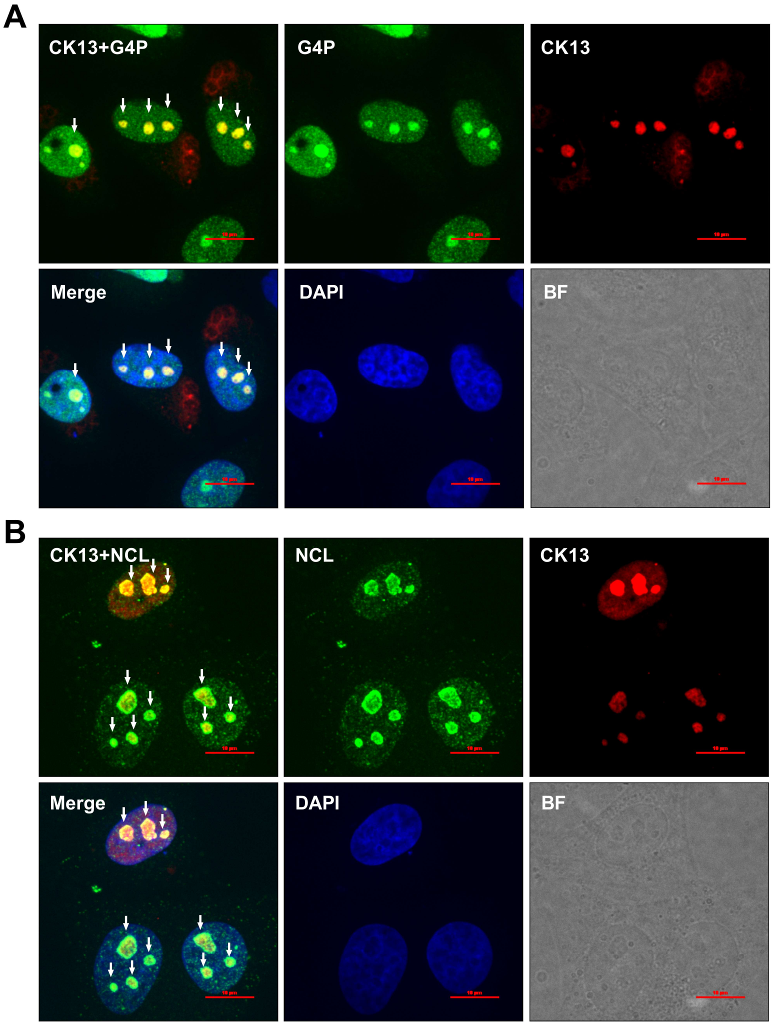

Figure S11. Colocalization of CK13 and G4-binding protein in cells. (A) Detection of the localization of CK13 and G4P in HeLa cells using immunofluorescence. HeLa cells were transiently transfected with plasmids encoding V5-tagged CK13 and FLAG-tagged G4P. The primary antibodies used were anti-V5 and anti-FLAG antibodies. CK13 foci were visualized with Alexa Fluor 555-conjugated antibody (red); G4P foci were visualized with Alexa Fluor 488-conjugated secondary antibody (green); Nucleus were stained with DAPI (blue); Merged images show colocalization as yellow signals; BF represents bright field image; Arrows denote colocalization sites. Scale bar: 10 μm. (B) Detection of the localization of CK13 and NCL in HeLa cells using immunofluorescence. HeLa cells were transiently transfected with plasmid encoding V5-tagged CK13. The primary antibodies used were anti-V5 and anti-Nucleolin antibodies. CK13 foci were visualized with Alexa Fluor 555-conjugated antibody (red); NCL foci were visualized with Alexa Fluor-488 conjugated secondary antibody (green); Nucleus were stained with DAPI (blue); Merged images show colocalization as yellow signals; BF represents bright field image; Arrows denote colocalization sites. Scale bar: 10 μm.

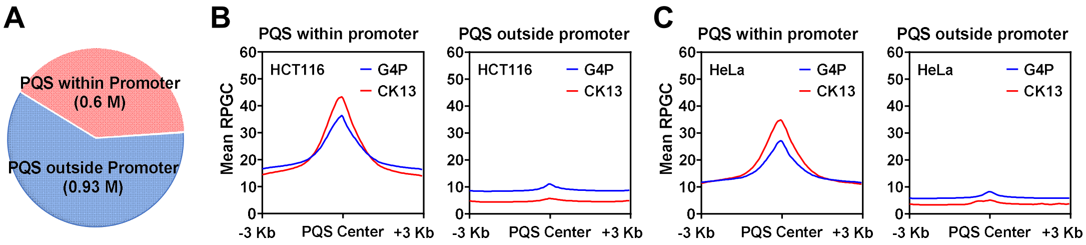

Figure S12. Enrichment of CK13 and G4P signals around the putative G4-forming sequences (PQS) in genomic DNA. (A) The number of PQS within and outside gene promoter regions (TSS ±3 kb). The bed file of PQS was obtained from the NCBI Gene Expression Omnibus (GEO) under accession code GSE133379. M represents the unit "million". (B-C) Signal profiles of CK13 and G4P enrichment across ±3 kb regions centered on PQS within and outside gene promoters in HCT116 and HeLa cells.

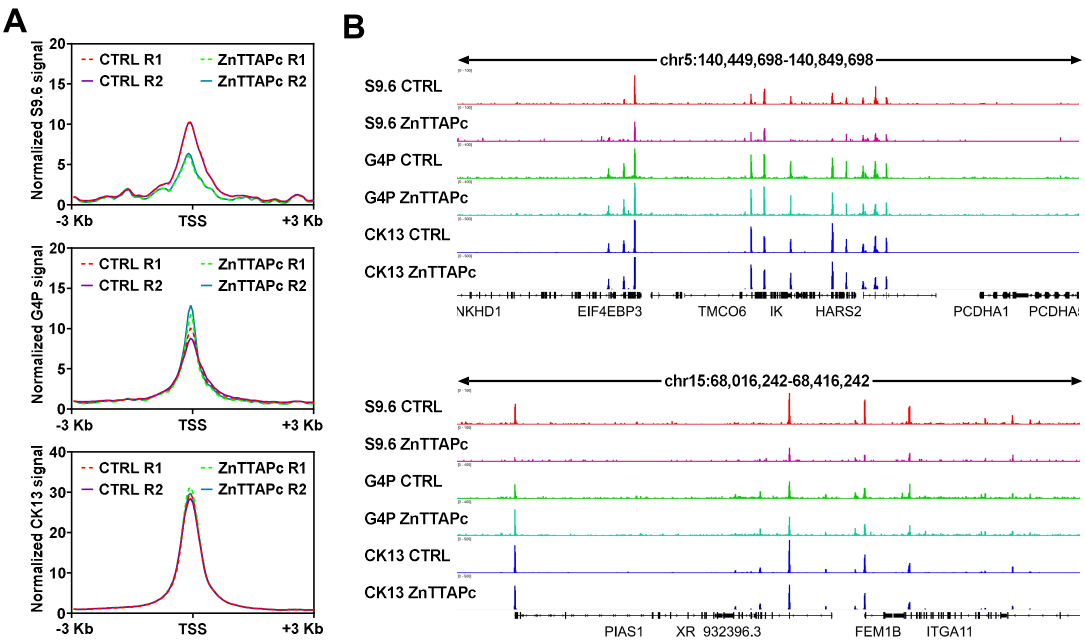

Figure S13. The binding signal of CK13 does not depend on R-loop formation. CUT&Tag data for R-loops were downloaded from NCBI GEO under accession number GSE239694. R-loop, G4P, and CK13 binding signals were analyzed in HCT116 cells treated or untreated with 10 μM ZnTTAPc using the CUT&Tag assay. (A) Distribution of R-loop (S9.6), G4P, and CK13 signals within the TSS ±3 kb regions. All signals are normalized by dividing by the signal intensity at the TSS −3 kb position. (B) Representative genomic loci showing R-loop formation sites (S9.6) and G4P and CK13 binding sites in HCT116 cells treated or untreated with 10 μM ZnTTAPc.

Table S1. Sequences of single-stranded DNAs used in EMSA and CD spectrum.

Name	Sequence (5′ to 3′)
3C	TTTTTTTTTTCCCTTTTTTTTTTT
4C	TTTTTTTTTTCCCCTTTTTTTTTT
5C	TTTTTTTTTCCCCCTTTTTTTTTT
6C	TTTTTTTTTCCCCCCTTTTTTTTT
7C	TTTTTTTTTCCCCCCCTTTTTTTT
(3-T1-3) C	TTTTTTTTTCCCTCCCTTTTTTTT
(2-T1-3) C	TTTTTTTTTCCTCCCTTTTTTTTT
(3-T3-3) C	TTTTTTTCCCTTTCCCTTTTTTTT
(3-T5-3) C	TTTTTTCCCTTTTTCCCTTTTTTT
(3-T7-3) C	TTTTTCCCTTTTTTTCCCTTTTTT
Tel-C4	CCCTAACCCTAACCCTAACCC
BCL2-C4	CCCGCCCCCTTCCTCCCGCGCCC
CSTB-C4	CCCCGCCCCGCGCCCCGCCCC
MYC-C4	CCCCACCCTCCCCACCC
MYC-C4-PC	CCCCACCCTCCCC-(PC linker)-ACCC
C9orf72-C4	CCCCGGCCCCGGCCCCGGCCCC
PDGFRB-1-C4	CCCCTGCCCCGCCGCCCTCCC
EGFR-C4	CTTCCTCCCGCCCTGCCTCCC
KRAS-C4	TTCCCTATTCCCACACCGCCCT
ABTB2-C4	CCCACTTCCCTCCGCCC
PDGFRB-2-C4	TGTCCCGCCGCCCTCCCTT
TBA-C4	CCAACCACACCAACC
KIT-1-C4	GCCGGCCACGCCCCTCCTCGCC

MYC-C124	CCCCACCCTTTTTACCC
MYC-C234	TTTTACCCTCCCCACCC
MYC-C14	CCCCATTTTTTTTACCC
MYC-C23	TTTTACCCTCCCCATTT
MYC-C3	TTTTATTTTCCCCATTT
MYC-C0	TTTTATTTTTTTTATTT
Tel-C124	CCCTAACCCTAATTTTAACCC
Tel-C234	TTTTAACCCTAACCCTAACCC
Tel-C12	CCCTAACCCTAATTTTAATTT
Tel-C13	CCCTAATTTTAACCCTAATTT
Tel-C2	TTTTAACCCTAATTTTAATTT
Tel-C0	TTTTAATTTTAATTTTAATTT
$(dA)_{24}$	AAAAAAAAAAAAAAAAAAAAAAAA
$(dT)_{24}$	TTTTTTTTTTTTTTTTTTTTTTTT
G4-forming sequence (BCL2)	GGGCGCGGGAGGAAGGGGGCGGG
G4-forming sequence (MYC)	GGGTGGGGAGGGTGGGG
G4-forming sequence (CSTB)	GGGGCGGGGCGCGGGGCGGGG
G4-forming sequence (Tel)	GGGTTAGGGTTAGGGTTAGGG
G4-forming sequence (KIT-2)	GGGCGGGCGCGAGGGAGGG
G4-forming sequence (C9orf72)	GGGGCCGGGGCCGGGGCCGGGG

Table S2. Sequences of single-stranded DNAs used in exonuclease digestion assay.

Name	Sequence (5′ to 3′)
Ls-Tel-C4	CCTGAAGCAGACAGCTAGTGAATTCCCCTTACCCTTACCCTTACCCTACTTGCGTATAACTGTTCCATAGT
Ls-MYC-C4	CCTGAAGCAGACAGCTAGTGAATTCCCCACCCTCCCCACCCCTTGCGTATAACTGTTCCATAGT

Tables S3. Sequences of dsDNAs used in EMSA.

<table>
<tr><th>Name</th><th>Sequences</th></tr>
<tr><td>dsBCL2</td><td>5′-ACTATGGAACAGTTATACGCAAGTACCCGCCCCCTTCCTCCCGCGCCCGAATTCACTAGCTGTCTGCTTCAGG-3′
|||||||||||||||||||||||||||||||||||||||||||||||||||||||||||||||||||||||||
3′-TGATACCTTGTCAATATGCGTTCATGGGCGGGGGAAGGAGGGCGCGGGCTTAAGTGATCGACAGACGAAGTCC-5′</td></tr>
<tr><td>dsKIT-2</td><td>5′-ACTATGGAACAGTTATACGCAAGTACCCTCCCTCGCGCCCGCCCGAATTCACTAGCTGTCTGCTTCAGG-3′
|||||||||||||||||||||||||||||||||||||||||||||||||||||||||||||||||||||
3′-TGATACCTTGTCAATATGCGTTCATGGGAGGGAGCGCGGGCGGGCTTAAGTGATCGACAGACGAAGTCC-5′</td></tr>
<tr><td>dsMYC</td><td>5′-ACTATGGAACAGTTATACGCAAGTACCCCACCCTCCCCACCCGAATTCACTAGCTGTCTGCTTCAGG-3′
|||||||||||||||||||||||||||||||||||||||||||||||||||||||||||||||||||
3′-TGATACCTTGTCAATATGCGTTCATGGGGTGGGAGGGGTGGGCTTAAGTGATCGACAGACGAAGTCC-5′</td></tr>
<tr><td>dsTel</td><td>5′-ACTATGGAACAGTTATACGCAAGTACCCTAACCCTAACCCTAACCCGAATTCACTAGCTGTCTGCTTCAGG-3′
|||||||||||||||||||||||||||||||||||||||||||||||||||||||||||||||||||||||
3′-TGATACCTTGTCAATATGCGTTCATGGGATTGGGATTGGGATTGGGCTTAAGTGATCGACAGACGAAGTCC-5′</td></tr>
<tr><td>Non-G4</td><td>5′-ACTATGGAACAGTTATACGCAAGAGTGTGCGTAGTGTGCGTAAATTCACTAGCTGTCTGCTTCAGG-3′
||||||||||||||||||||||||||||||||||||||||||||||||||||||||||||||||||
3′-TGATACCTTGTCAATATGCGTTCTCACACGCATCACACGCATTTAAGTGATCGACAGACGAAGTCC-5′</td></tr>
</table>

Table S4. Amino acid sequences of the protein probes used in this study. The blue font indicates the KH1 domain of PCBP1, the green font indicates the KH3 domain of hnRNPK, and the red font indicates the V5-tag.

Name	Amino acid sequence (N to C)
C1	ISHMLTIRLLMHGKEVGSIIGKKGESVKRIREESGARINISEGNCPERIITLTGPTNAIFKAFAMIIDKLEED<br>YGTGSGAGKPIPNPLLGLDST
K3	ISHMGGPIITTQVTIPKDLAGSIIGKGGQRIKQIRHESGASIKIDEPLEGSEDRIITITGTQDQIQNAQYLLQ<br>NSVKQYGTGSGAGKPIPNPLLGLDST
diC1	ISHMLTIRLLMHGKEVGSIIGKKGESVKRIREESGARINISEGNCPERIITLTGPTNAIFKAFAMIIDKLEED<br>YGTGSGAMLTIRLLMHGKEVGSIIGKKGESVKRIREESGARINISEGNCPERIITLTGPTNAIFKAFAMIID<br>KLEEDYGTGSGAGKPIPNPLLGLDST
diK3	ISHMGGPIITTQVTIPKDLAGSIIGKGGQRIKQIRHESGASIKIDEPLEGSEDRIITITGTQDQIQNAQYLLQ<br>NSVKQYINSSMTNSTAASGGPIITTQVTIPKDLAGSIIGKGGQRIKQIRHESGASIKIDEPLEGSEDRIITIT<br>GTQDQIQNAQYLLQNSVKQYGTGSGAGKPIPNPLLGLDST
CK13	ISHMLTIRLLMHGKEVGSIIGKKGESVKRIREESGARINISEGNCPERIITLTGPTNAIFKAFAMIIDKLEEDI<br>NSSMTNSTAASGGPIITTQVTIPKDLAGSIIGKGGQRIKQIRHESGASIKIDEPLEGSEDRIITITGTQDQIQ<br>NAQYLLQNSVKQYGTGSGAGKPIPNPLLGLDST

Reference

1. Zheng, K.W., Chen, Z., Hao, Y.H. and Tan, Z. (2010) Molecular crowding creates an essential environment for the formation of stable G-quadruplexes in long double-stranded DNA. *Nucleic Acids Res*, **38**, 327-338.
